# The effect of provenance and species on the chemical composition of *Epilobium* herbal tea

**DOI:** 10.1080/13880209.2026.2672676

**Published:** 2026-05-18

**Authors:** Olha Mykhailenko, Banaz Jalil, Kateryna Uminska, Liudas Ivanauskas, Zigmantas Gudžinskas, Michael Heinrich

**Affiliations:** a Pharmacognosy and Phytotherapy Group, UCL School of Pharmacy, London, United Kingdom; b National University of Pharmacy, Kharkiv, Ukraine; c Zhytomyr Basic Pharmaceutical Professional College, Zhytomyr, Ukraine; d Department of Analytical and Toxicological Chemistry, Lithuanian University of Health Sciences, Kaunas, Lithuania; e Laboratory of Flora and Geobotany, State Scientific Research Institute Nature Research Centre, Vilnius, Lithuania; f Department of Pharmaceutical Sciences and Chinese Medicine Resources and Chinese Medicine Research Center, College of Chinese Medicine, China Medical University, Taichung, Taiwan

**Keywords:** Polyphenolic compounds, oenothein B, quality markers, herb MaRS, PAINS

## Abstract

**Background:**

Commercial herbal teas marketed as *Epilobium* herb are widely consumed in Europe for prostate-related and anti-inflammatory effects. However, they are often sold under generic names without clear species differentiation or quality specifications, raising concerns about chemical consistency and therapeutic equivalence.

**Objective:**

To evaluate the chemical equivalence of commercial *Epilobium* products and refine the framework for quality marker selection by integrating pharmacological relevance with structural assessment.

**Materials and Methods:**

Sixteen commercial tea samples labeled as *E. angustifolium, E. parviflorum, E. hirsutum*, from eight European countries were analyzed using high-performance thin-layer chromatography (HPTLC) and validated by HPLC–DAD. Candidate markers were prioritized using the Herbal Chemical Marker Ranking System (Herb MaRS) and further assessed *via* Pan-Assay Interference Compounds (PAINS) structural filters.

**Results:**

Significant qualitative and quantitative heterogeneity was observed across all samples, including those labeled as the same species. Oenothein B was the dominant compound but exhibited high variability (7.9–48.7 mg/g DW; CV >50%) and was significantly higher in *E. hirsutum* (*p* < 0.05). Flavonol glycosides (e.g., hyperoside, isoquercitrin) also varied substantially. Although species identity influenced phenolic profiles, overlapping concentration ranges indicated a lack of chemical equivalence among products. Geographic origin contributed additional but secondary variability.

**Discussion:**

Oenothein B, hyperoside, and isoquercitrin were identified as priority quality markers based on abundance and analytical suitability. Structural reassessment demonstrated that pharmacological ranking alone may be misleading for polyphenol-rich matrices due to redox-related interference.

**Conclusion:**

Commercial *Epilobium* teas differ chemically and are not interchangeable. A structure-informed approach is essential for reliable quality assessment and for aligning commercial products with pharmacopeial standards.

## GRAPHICAL ABSTRACT

**Figure:**
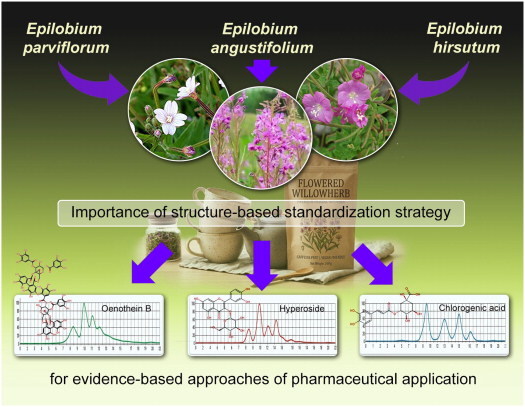

## Introduction

Species of the genus *Epilobium* L. (Onagraceae), particularly *E. angustifolium* L., *E. parviflorum* Schreb. and *E. hirsutum* L., have a long history of use in traditional medicine across Europe, Asia, and North America (Wagner et al. 2007; Kalle et al. 2020; Sõukand et al. 2020). In Central and Eastern Europe, several *Epilobium* species have traditionally been used to make herbal teas for various purposes. *E. angustifolium* and *E. parviflorum* are most often used for their potential benefits as preventive and treatment options for benign prostate hyperplasia (BPH), urinary tract disorders, gastrointestinal disorders, skin conditions, and as a tea for fatigue, colds, and insomnia (Ducrey et al. 1997; Stolarczyk M, Piwowarski JP, et al. 2013).

*Epilobium parviflorum* (commonly known as small-flowered willowherb) was particularly popular in traditional Austrian medicine (EMA/HMPC/712510/2014 2015). Infusions or teas made from the plant were used to treat disorders of the genitourinary system, such as problems with the prostate gland, bladder and kidneys. The extracts have also been shown to possess anti-inflammatory and antioxidant properties in experiments (Adamczak et al. 2019).

*Epilobium angustifolium* (commonly known as fireweed, rosebay willowherb) is widely consumed as a caffeine-free herbal tea. It is often fermented to enhance aroma and flavor, and it is particularly popular in Ukraine and the Nordic countries. There, it has long been considered a general tonic and a remedy for inflammation and gastrointestinal disorders (Granica et al. 2014; Lasinskas et al. 2020). In recent decades, *Epilobium*-based herbal tea has become popular as a potential remedy for insomnia, for improving digestion, and for treating prostate cancer (Kalle et al. 2020; Sõukand et al. 2020).

*Epilobium hirsutum* (commonly known as great hairy willowherb) has been used less frequently in traditional medicine, but ethnobotanical records report its use as a topical anti-inflammatory and wound-healing agent (Sõukand et al. 2020). In eastern and northeastern Europe (Poland, Ukraine, Lithuania, Latvia, Estonia), the leaves of *E. hirsutum* are commonly consumed as tea to treat gastrointestinal ailments (Granica et al. 2014).

In the European Union, herbal teas are generally classified as foods (i.e., herbal infusions), unless they are registered as traditional herbal medicinal products under the Directive 2004/24/EC. Therefore, the commercial samples included and analyzed here are available on the market as herbal tea, although the plant species used in these products may also possess potential medicinal properties.

Modern pharmacological research provides experimental support for the traditional use of *Epilobium* species, particularly regarding their anti-inflammatory (Nowak et al. 2022), antioxidant (Ivanauskas et al. 2024), antimicrobial (Battinelli et al. 2001), and antiproliferative properties. These properties have been demonstrated in cell-based models, including prostate cell models (Vitalone et al. 2003). These activities are primarily attributed to the presence of polyphenolic compounds such as ellagitannins (e.g., oenothein A and B), flavonoids (e.g., quercetin derivatives including hyperoside, isoquercitrin, avicularin, guaijaverin) and phenolic acids (e.g., gallic acid, chlorogenic acid) (Agnieszka et al. 2018; Mykhailenko et al. 2025). Ellagitannins have shown inhibitory activity against 5α-reductase, which is a relevant mechanism for managing BPH (Ducrey et al. 1997; Kiss et al. 2004). However, most of this pharmacological data is derived from *in vitro* or preclinical studies using isolated compounds or enriched extracts. Clinical data remain limited, and the available evidence does not yet provide a sufficient basis to support general therapeutic claims for commercially available *Epilobium* products.

Studies on the chemical profiling of *Epilobium* species have demonstrated that the content of these bioactive compounds is highly variable and influenced by multiple factors, including species identity, geographical origin, harvest time, which plant organs are used and how they are processed (Monschein et al. 2015; Baert et al. 2017). This variability makes it difficult to ensure the consistent quality and efficacy of commercial herbal products, especially when they are marketed under the same common name without specifying the species or geographical origin.

Despite extensive phytochemical and pharmacological research on *Epilobium* species, there remains a significant discrepancy among their traditional uses, available chemical knowledge, evidence-based recommendations for commercial products, and approaches to quality control of plant raw materials. *Epilobium* plant material is widely available on the European pharmaceutical market as herbal teas and food supplements (Stolarczyk et al. 2013). However, products marketed as herbal teas and/or food supplements are problematic in how their pharmacological relevance and quality requirements are interpreted (Jalil and Heinrich 2025). *E. angustifolium* and *E. parviflorum* are described in EMA/ESCOP monographs on medicinal plants, with a focus on traditional pharmacological uses and safety (EMA/HMPC/712510/2014 2015; EMA/HMPC/712511/2014 2015, ESCOP 2024). While the United States Pharmacopeia and the Chinese Pharmacopeia lack specific pharmacopeial monographs for *Epilobium* species, herbal teas are widely available on the market (Epilobium Inc. 2026; BiovitaFusion, 2026). However, available monographs do not describe chemical quality control of the herbal product or the associated quantitative variability of key phenolic components. As a result, recognition of traditional uses by regulatory authorities does not necessarily guarantee chemical equivalence of commercially available products.

Herbal teas and supplements derived from different *Epilobium* species and geographical origins are often marketed under a common name, without clear species identification, quantitative quality specifications, or standardized quality control. This raises several questions about the consistency, safety, and scientific justification of generalized health-related claims and recommended intake levels for such products, which are conceptually distinct from medicinal/medical products (Heinrich et al. 2022). For food products such as herbal teas, health claims and intake levels are often implied rather than formally substantiated under the applicable food regulations (e.g., EU Regulations No. 1924/2006 on nutrition and health claims). In other words, the same plant species with different chemical profiles or differing quantities of pharmacologically active metabolites may not be comparable in terms of composition and potential effects.

In addition, many pharmacological results regarding *Epilobium* species are based primarily on *in vitro* studies using polyphenol-rich extracts. This, in turn, may overstate biological significance and does not necessarily translate into clinically significant effects.

The present study aims to assess and evaluate the chemical profiles of commercial *E. angustifolium*, *E. parviflorum,* and *E. hirsutum* products originating from different countries using High-Performance Thin-Layer Chromatography (HPTLC) and High-Performance Liquid Chromatography (HPLC) in combination with the Herbal Chemical Marker Ranking System (Herb MaRS) (Bensoussan et al. 2015), as well as Pan-Assay Interference Compounds (PAINS) filter to justify the choice of optimal quality markers for herbal products. By identifying and quantifying key polyphenolic quality markers, the study seeks to assess inter-sample variability and establish the chemical and pharmacological relevance of commercial *Epilobium* products marketed under the same common name, thereby emphasizing the importance of species- and origin-specific quality control.

Despite numerous studies on the phytochemical composition of wild and cultivated *Epilobium* species, systematic comparative analyses of commercial *Epilobium* teas from different species and countries of origin still remain rare. There is a lack of information on whether products sold under the same scientific or common name have the same polyphenol content, which is important for pharmacology.

The following research questions were posed: (a) How do the composition and content of active metabolites differ between *Epilobium* herbal teas originating from the same species but different countries? (b) What are the differences in the composition and quantity of biologically active metabolites between *Epilobium* species? (c) Which marker compounds are most suitable for further quality assessment?

## Material and methods

### Commercial plant sample

**Figure 1.**
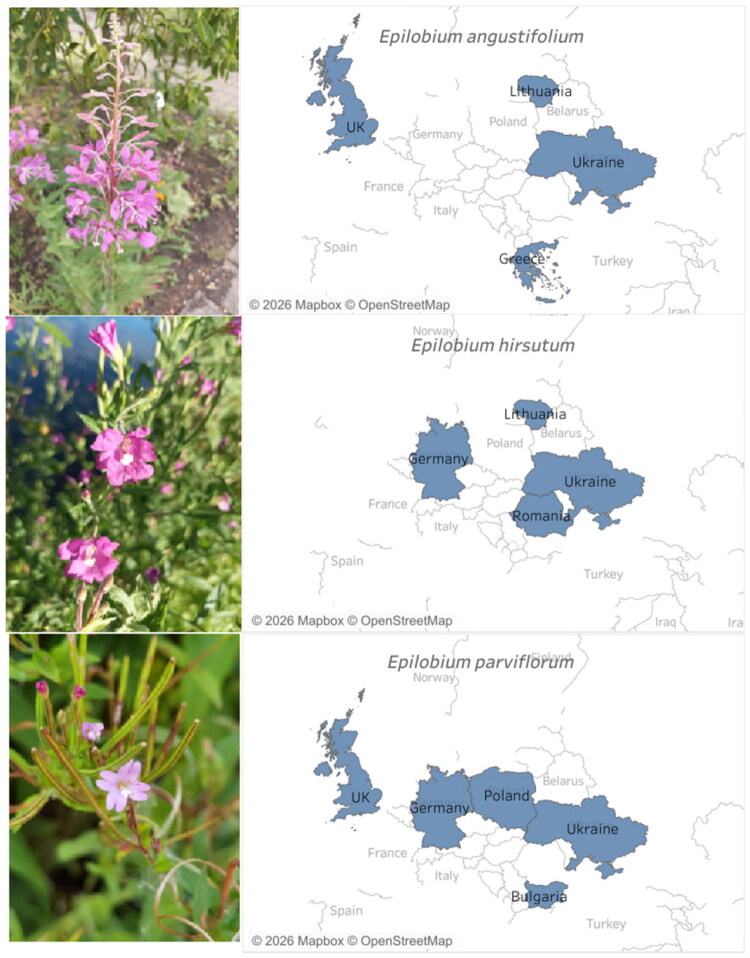
*Epilobium angustifolium*, *Epilobium hirsutum* and *Epilobium parviflorum*, as well as the countries of origin of the analyzed commercial samples. Photos of the plants were taken at the botanical garden of Kiel University in July 2025. Photographs by O. Mykhailenko.

Commercial *Epilobium* products marketed as herbal teas were purchased from retail outlets and online sources in various European countries. The products were selected based on their labeling as *Epilobium* tea, and included samples declared as *E. angustifolium, E. parviflorum, E. hirsutum*, as well as samples for which the species was not specified. All samples were analyzed as marketed, without additional quantification, to reflect variability between commercial products (Table 1, Figure 1**;** Fig. S1). Brand names and manufacturers are not disclosed in the manuscript to avoid commercial bias, but this information is available from the corresponding author upon reasonable request.

**Table 1.** List of commercial herb samples of *Epilobium* species used in the analysis.

Code	*Epilobium* species	Country	Country code
CS-1	*E. parviflorum*	Poland	PL
CS-2	*E. parviflorum*	Bulgaria	BG
CS-3	*E. parviflorum*	Germany	DE
CS-4	*E. parviflorum*	United Kingdom	GB
CS-5	*E. parviflorum*	United Kingdom	GB
CS-6	*E. parviflorum*	Ukraine	UA
CS-7	*E. hirsutum*	Romania	RO
CS-8	*E. hirsutum*	Germany	DE
CS-9	*E. hirsutum*	Ukraine	UA
CS-10	*E. hirsutum*	Lithuania	LT
CS-11	*E. angustifolium*	Greece	GR
CS-12	*E. angustifolium*	Greece	GR
CS-13	*E. angustifolium*	United Kingdom	GB
CS-14	*E. angustifolium*	United Kingdom	GB
CS-15	*E. angustifolium*	Ukraine	UA
CS-16	*E. angustifolium*	Lithuania	LT

### Chemicals and reference standards

The reference standards for gallic acid, ellagic acid, chlorogenic acid, caffeic acid, oenothein B, hyperoside, isoquercitrin, avicularin, guaijaverin, afzelin, rutin, kaempferol, quercetin, and myricetin were obtained from ChromaDex Inc. (Santa Ana, USA) and Sigma-Aldrich (Saint Louis, USA). All solvents (acetonitrile, methanol and glacial acetic acid) were of HPLC grade and were purchased from Merck KGaA, Fisher Scientific Ltd. and VWR International LLC. Deuterated methanol, which was purchased from Cambridge Isotope Laboratories Inc. Analytical- and chromatographic-grade chemicals and solvents were used for this study, including acetonitrile, methanol and glacial acetic acid, all from Sigma-Aldrich GmbH (Karlsruhe, Germany). Water was purified using a ULTRAPURE water system (Millipore, Germany). All other chemicals were of analytical grade.

### Sample preparation

The extraction procedure was optimized for phytochemical profiling and does not directly represent consumer-prepared aqueous infusions. The sample preparation process was described in detail by Mykhailenko et al. (2025). For the HPTLC analysis, 0.50 g of air-dried, powdered plant material from each sample was extracted with 50% (v/v) methanol (1:10) using ultrasound-assisted extraction for 20 min at 40 °C. For the HPLC analysis, 0.20 g of powder from each sample was extracted in 10 mL of 50% (v/v) methanol in an ultrasonic bath (WiseClean) at 45 ± 2 °C for 20 min. The extracts were filtered using a Millex R syringe filter unit with 0.22 µm pore size. The reference standards (avicularin, guaijaverin, hyperoside, oenothein B, isoquercitrin, gallic acid, ellagic acid, chlorogenic acid, and myricetin) were dissolved in methanol at a concentration of 1 mg/mL and sonicated for 10 min. Both the reference standards and the test samples were stored at 4 °C. Injection volumes of 3 μL of reference standards and 7 μL of sample solutions were used.

### HPTLC analysis

HPTLC was used to visualize inter-sample variability and support multivariate comparison of commercial products. The samples and reference standards were applied using a CAMAG Linomat 5 semi-automated sampler. The HPTLC glass plates, silica gel 60 F_254_ used for the stationary phase, were purchased from Merck KGaA (Darmstadt, Germany). The samples and reference standards were applied to the plates as 8 mm bands using a 100 μL CAMAG syringe under a gentle nitrogen flow, with the following parameters: 15 mm from the left edge, 10 mm from the lower edge, and 10 mm track spacing.

The plates were developed in the ADC 2 with chamber saturation (using filter paper) for 20 min. After activation at 33% relative humidity for 10 min using a saturated magnesium chloride solution, development continued with ethyl acetate, formic acid, and water (68:8:8 *v/v*) until the migration distance reached 70 mm from the lower edge. The plates were then dried for 5 min. The temperature and relative humidity were controlled to 21–24 °C and 33%, respectively.

After development, the plates were derivatised using the Natural Product A/PEG reagent system. They were then heated at 100 °C on a CAMAG TLC plate heater for 5 min to enhance the visualization of phenolic compounds. The plates were then visualized using a CAMAG Visualizer under white and UV light at 254 nm and 366 nm. The resulting chromatographic fingerprints were documented and compared using VisionCATS version 3.1 software.

### HPLC-DAD quantification

Chromatographic conditions were adapted from previously validated methods, with minor modifications to ensure the optimal separation of the selected quality markers in *Epilobium* samples. Polyphenol separation was performed using a Waters e2695 Alliance HPLC system coupled with a 2998 PDA detector (Waters, Milford, MA, USA). Phenolic compounds were separated on an ACE Super C_18_ (250 mm × 4.6 mm, 3 µm) column (ACT, Aberdeen, UK) with a full run time of 81 min. Column temperature was 25 °C. The gradient elution mode consisting of 0.1% (*v*/*v*) trifluoroacetic acid in pure water (A) and acetonitrile (B) was as follows: 0 min, 5% B; 8–30 min, 20% B; 30–48 min, 40% B; 48–58 min, 50% B; 58–65 min, 50% B; 65–66 min, 95% B; 66–70 min, 95% B; 70–81 min, 5% B. The flow rate was 1.000 mL/min, and the injection volume was 10 µL. Tannins were detected at 219 nm, and flavonoids and phenolic acids at 350 nm (maximum absorption), with peaks identified by comparison of retention times with standards (Table S1). The samples were subjected to two different analyses. The quantity of oenothein A was obtained by recalculating the quantity of the calibration curve of oenothein B, and the quantity of myricetin-3-O-glucoside (isomyricitrin) was obtained by recalculating the quantity of the calibration curve of myricetin. The analytical HPLC-PDA method for polyphenols was validated according to the ICH Q2(R1) guidelines and was described by Ivanauskas et al. (2024).

### Selection of quality markers

Initially, quality markers were prioritized using the Herbal Chemical Marker Ranking System (Herb MaRS) (Bensoussan et al. 2015), which was employed to identify polyphenolic compounds reported in *Epilobium* species for the comparative quality assessment of commercial herbal products. Herb MaRS integrates multiple criteria, including documented *in vitro*, *in vivo,* and clinical biological activity data; relevance to traditional or current therapeutic use; the typical concentration of the herbal material; the availability of reliable reference standards; and safety or toxicity considerations.

The pharmacological effects of compounds associated with prostate-related disorders and with anti-inflammatory and antioxidant activities were evaluated in the context of *Epilobium* species. According to the Herb MaRS criteria, candidate markers are ranked on a scale from 0 (least suitable) to 5 (the highest priority). A score of 5 is assigned to compounds demonstrating high pharmacological relevance, sufficient concentrations (≥ 5 µg/g), evidence of bioavailability, or requiring monitoring due to potential toxicity. This must be supported by at least two high-quality publications.

The major compounds of *Epilobium* species were assessed using the following sources: published literature, the PubChem BioAssay Database, the Cochrane Library, and available compositional data for herbal raw materials. The preliminary ranking and selected candidate markers are summarized in Table S2.

Compounds that received higher Herb MaRS scores were selected for quantitative analysis and univariate statistical testing. Lower-ranked compounds were retained to support chromatographic fingerprinting and the multivariate evaluation of overall chemical variability (Kaggwa et al. 2023).

Although Herb MaRS provides a structured and rational framework for prioritizing candidate quality markers, the workflow does not explicitly address structural features that contribute to assay interference. Therefore, an additional assessment step has been introduced to reevaluate the chemical robustness of the selected candidates before they are confirmed as pharmacologically meaningful quality markers.

The selection of quality markers was based on their suitability for quality control, taking into account analytical detectability and pharmacological relevance, rather than on their use as chemotaxonomic markers.

### Detection of pan assay interference compounds (PAINS)

A second-stage assessment step focusing on Pan-Assay Interference Compounds (PAINS) was implemented (Baell and Holloway 2010; Magalhães et al. 2021). This assessment was conducted to refine the preliminary Herb MaRS-based selection and as markers of pharmacological relevance within quality assessment.

All selected Herb MaRS candidates underwent *in silico* analysis using three publicly available PAINS filters from the ChEMBL, RDKit, and SwissADME web tools (Daina et al. 2017). Canonical SMILES strings were retrieved from the PubChem database and used as input for the analysis (Table S3). PAINS (Baell and Holloway 2010) and Brenk structural alerts (Brenk et al. 2008) were used to identify substructures that could cause nonspecific assay interference. This includes reactive or redox-active moieties that could lead to false-positive results in certain *in vitro* bioassays. Redox risk was qualitatively assessed based on the presence of catechol or pyrogallol structural moieties. PubChem BioAssay density was estimated from the number of reported bioassays, providing context for reported biological activity. These descriptors were used to provide interpretative support rather than for numerical scoring.

The use of PAINS- predisposed compounds as quality markers may bias the pharmacological interpretation and quality assessment of commercial products. In this context, “quality” refers not only to analytical suitability (e.g., detectability and reproducibility) but also to the reliability of the pharmacological interpretation of marker compounds. Therefore, PAINS screening was not used as an automatic exclusion criterion, but rather as an additional step to critically reevaluate the structural suitability of candidate markers within the overall workflow. To confirm the reliability of the *in silico* results, a review of published *in vivo* studies reporting anti-inflammatory and related biological activities of individual compounds was conducted.

### Statistical analysis

The data distribution in the datasets was assessed using the Shapiro–Wilk test. As all data were non-normally distributed, non-parametric statistical analysis methods were employed. The significance level was set at *p* < 0.05. The Kruskal-Wallis test was used to detect differences between sets of samples, and the Mann-Whitney test was used for pairwise comparisons between samples. Spearman’s rank correlation analysis (r_s_) was performed in PAST v.5.2.2 (Hammer et al. 2001) to investigate the relationship between the selected phenolic compounds and support the multivariate structure of the dataset. All data were processed using the Waters^®^ Empower 3 software and Microsoft Excel 2010. Results are presented as the mean ± standard deviation (SD) of three replicates. Mean values and coefficients of variation (CV, expressed as a percentage) were calculated for each compound across all commercial samples for each compound using absolute cell references in Excel (CV = SD/mean × 100).

Data visualization was performed using Tableau Desktop Public Edition 2025.3.1 (Tableau Software, USA). The mean compound content (mg/g) of *Epilobium* species was compared using grouped bar charts with standard deviation.

## Results

### Qualitative variability of commercial Epilobium species

**Figure 2.**
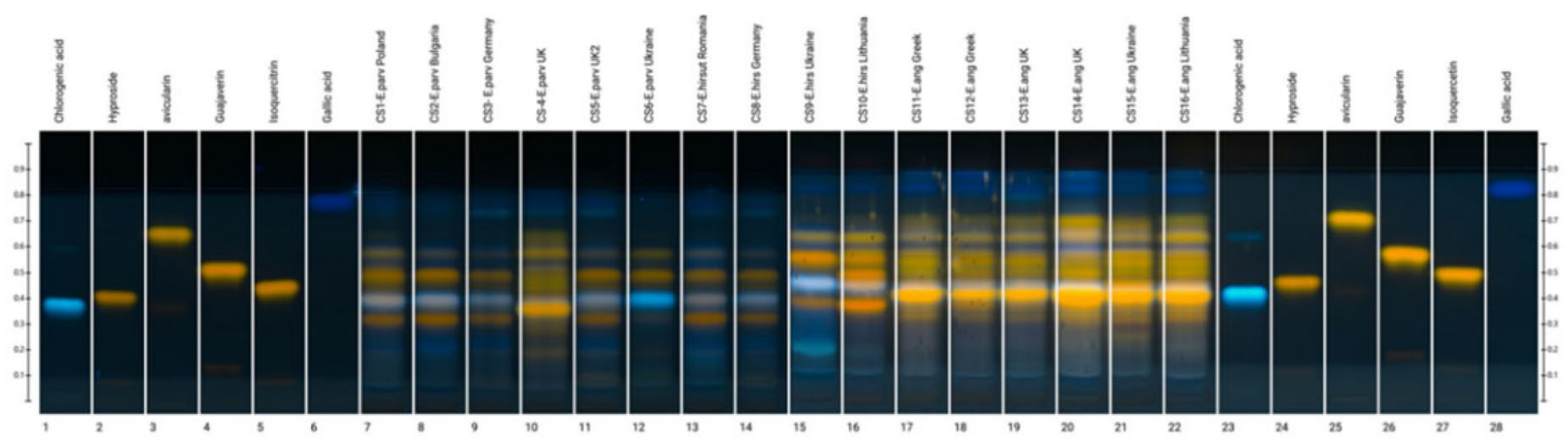
HPTLC analysis of commercial samples of *Epilobium* species, following derivatisation at UV 366 nm. System: ethanol: formic acid: water (68:8:8). Derivatisation was performed using diphenylboric acid aminoethyl ester, followed by macrogol 400 and heating at 105 °C for 5 min.

HPTLC analysis was used to assess chemical differences and similarities among products claimed to be derived from the same *Epilobium species*, as well as to visualize qualitative differences in polyphenolic profiles and the overall diversity of the products. HPTLC fingerprints revealed qualitative and semi-quantitative differences among commercial samples, including those labeled as the same *Epilobium* species (Figure 2).

The main differences observed were in the flavonoid and ellagitannin zones (e.g., at R_f_ values) among samples labeled as the same species. Although all analyzed samples exhibited characteristic phenolic zones corresponding to flavonoid glycosides and ellagitannins, the relative intensities of their bands and their overall fingerprint patterns varied significantly.

Among the samples labeled as *E. parviflorum*, the products originating from Poland (CS-1), Bulgaria (CS-2), and Germany (CS-3) had similar chromatographic profiles. These were characterized by zones of moderate intensity corresponding to flavonoid glycosides. By contrast, the sample labeled as *E. parviflorum* from the United Kingdom (CS-4) had a markedly different profile, showing a dominant yellow, fluorescent zone at an R_f_ value of approximately 0.37. This corresponded in color and migration distance to the hyperoside reference standard. This profile was similar to those observed for several samples labeled as *E. angustifolium* (CS-11 to CS-15), rather than the other *E. parviflorum* products.

Commercial samples labeled as *E. angustifolium* exhibited relatively consistent qualitative profiles across different countries of origin. These profiles were characterized by zones corresponding to hyperoside (R*_f_* ≈ 0.37), isoquercitrin (R*_f_* ≈ 0.40), avicularin (R*_f_* ≈ 0.52) and guaijaverin (R*_f_* ≈ 0.85). However, notable differences in band intensity were observed among individual products, indicating variability in the relative abundances of these compounds, despite overall similarity in fingerprint composition.

Samples labeled as *E. hirsutum* exhibited significant heterogeneity in their HPTLC profiles. While the presence of major flavonoid glycosides was confirmed in all *E. hirsutum* samples, the intensity of the bands corresponding to ellagitannins and flavonoids varied significantly between products from different countries. This indicates inconsistent levels of key phenolic components.

The reference standards gallic acid, chlorogenic acid, hyperoside, isoquercitrin, avicularin, and guaijaverin were identified in all analyzed samples, confirming their widespread occurrence in commercial *Epilobium* products (Fig. S2). However, the observed variability in chromatogram patterns and band intensity suggests that products marketed under the same species name are not chemically equivalent at a qualitative level.

Overall, HPTLC analysis revealed significant variability among commercial *Epilobium* teas, emphasizing inconsistencies within and between products labeled as a specific species.

### Quantitative variability of phenolic compounds in Epilobium species

**Figure 3.**
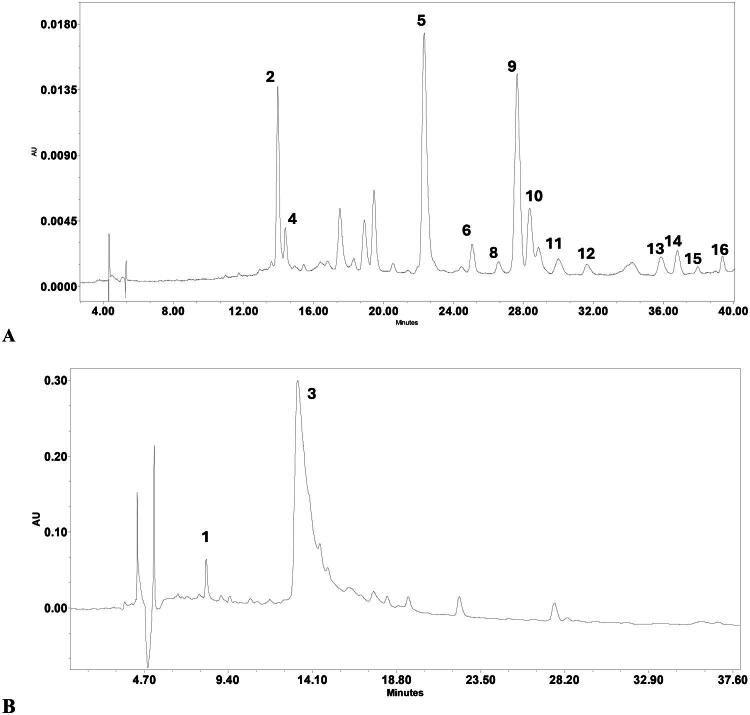
HPLC-DAD Chromatograms of the 50% methanol extracts of *Epilobium parviflorum* (CS3). Chromatograms were recorded at 350 nm (A) for identified polyphenols and at 219 nm (B) for tannins. Peaks: 1 – gallic acid; 2 – chlorogenic acid; 3 – oenothein B; 4 – oenothein A; 5 – myricetin glucoside; 6 – ellagic acid; 7 – rutin; 8 – hyperoside; 9 – isoquercitrin; 10 – guaijaverin; 11 – avicularin; 12 – quercitrin; 13 – myricetin; 14 – afzelin; 15 – kaempferol; 16 – quercetin.

To assess variability in the concentrations of key compounds among samples, a quantitative HPLC-DAD analysis (Figure 3, Fig. S3, Fig. S4) was performed, focusing on the major phenolic compounds in commercial *Epilobium* samples. This analysis was performed to complement the qualitative differences observed by HPTLC fingerprinting (Figure 2). Sixteen commercial samples labeled as *E. angustifolium, E. hirsutum*, or *E. parviflorum* from European countries (Poland, Bulgaria, Germany, the United Kingdom, Ukraine, Romania, Lithuania, and Greece) were analyzed for ellagitannins, flavonoid glycosides, and phenolic acids.

**Figure 4.**
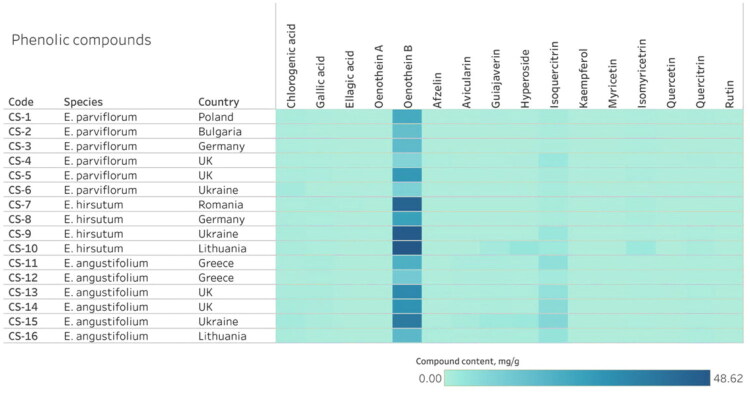
The phytochemical profile heatmap of 16 commercial *Epilobium* samples from different countries. The color intensity indicates the mean concentration (in mg/g DW) of individual phenolic compounds, revealing the distribution patterns and variability among the samples.

Across all samples, ellagitannins and flavonols were the dominant phenolic classes. Oenothein B was the most abundant individual compound in most of the samples (Figure 4, Fig. S6). Significant quantitative variability was found both between and within species, indicating significant chemical heterogeneity among samples.

To evaluate the chemical distinctiveness of the species, the quantitative data were grouped by species (*E. parviflorum, E. hirsutum, E. angustifolium*), and the mean concentrations of the main phenolic compounds were compared using non-parametric statistical tests (Table 2).

**Table 2.** The content of phenolic compounds (mg/g DW; mean ± SD, standard deviation) in the raw material of the studied *Epilobium* species.

Peak on Figure 3, S3, S4	RT, min	Selected wavelengths (λ,, nm) for quantitative determination of compounds	Compound	*E. parviflorum* (*n* = 6)	*E. hirsutum* (*n* = 4)	*E. angustifolium* (*n* = 6)
1	8.10	274	Gallic acid	0.335 ± 0.178^a^	0.560 ± 0.068^b^	0.640 ± 0.216^b^
3	14.50	265	Oenothein B	16.508 ± 7.595^a^	41.417 ± 10.065^b^	24.817 ± 9.357^c^
2	13.60	324	Chlorogenic acid	0.753 ± 0.390^a^	0.730 ± 0.168^a^	0.896 ± 0.405^a^
4	15.04	265	Oenothein A	0.045 ± 0.014^a^	0.072 ± 0.038^b^	0.074 ± 0.019^b^
5	22.41	360	Myricetin glucoside	0.434 ± 0.310^a^	1.237 ± 0.975^b^	0.295 ± 0.273^a^
6	22.48	254	Ellagic acid	0.126 ± 0.047^a^	0.251 ± 0.149^a^	0.057 ± 0.023^b^
7	26.66	350	Rutin	0.001 ± 0.000^a^	0.001 ± 0.000^a^	0.187 ± 0.127^b^
8	26.64	355	Hyperoside	0.157 ± 0.131^a^	0.906 ± 1.474^a^	0.799 ± 0.782^b^
9	27.68	355	Isoquercitrin	1.267 ± 0.652^a^	1.767 ± 0.850^a^	4.678 ± 1.930^b^
10	28.31	360	Guaijaverin	0.153 ± 0.152^a^	0.478 ± 0.706^b^	0.850 ± 0.691^c^
11	30.79	256	Avicularin	0.059 ± 0.080^a^	0.060 ± 0.053^a^	0.548 ± 0.342^b^
12	31.04	355	Quercitrin	0.260 ± 0.131^a^	0.478 ± 0.357^ac^	0.358 ± 0.129^bc^
13	35.67	360	Myricetin	0.052 ± 0.033^a^	0.127 ± 0.108^b^	0.021 ± 0.011^c^
14	37.95	360	Afzelin	0.138 ± 0.195^a^	0.184 ± 0.371^a^	0.304 ± 0.206^b^
15	38.93	360	Kaempferol	0.010 ± 0.002^a^	0.021 ± 0.020^a^	0.013 ± 0.008^a^
16	40.65	360	Quercetin	0.017 ± 0.008^a^	0.016 ± 0.009^a^	0.040 ± 0.019^b^

Different superscript letters in the same row indicate significant differences (*p* < 0.05) in the quantity of a given compound, according to the results of the Mann–Whitney pairwise comparison.

#### Ellagitannins

The total content of phenolic compounds varied widely, with ellagitannins (oenothein A and B) and flavonols (hyperoside, isoquercitrin) predominating (Figure 4, Fig. S6). The heatmap (Figure 4, Fig. S5) shows that oenothein B is the dominant compound in all species, with contents ranging from 7.9 to 48.7 mg/g DW in individual samples. Additional bar charts (Figure 6, Figure 7) show species-dependent variation in flavonoid and hydroxycinnamic acid content, excluding oenothein B.

**Figure 5.**
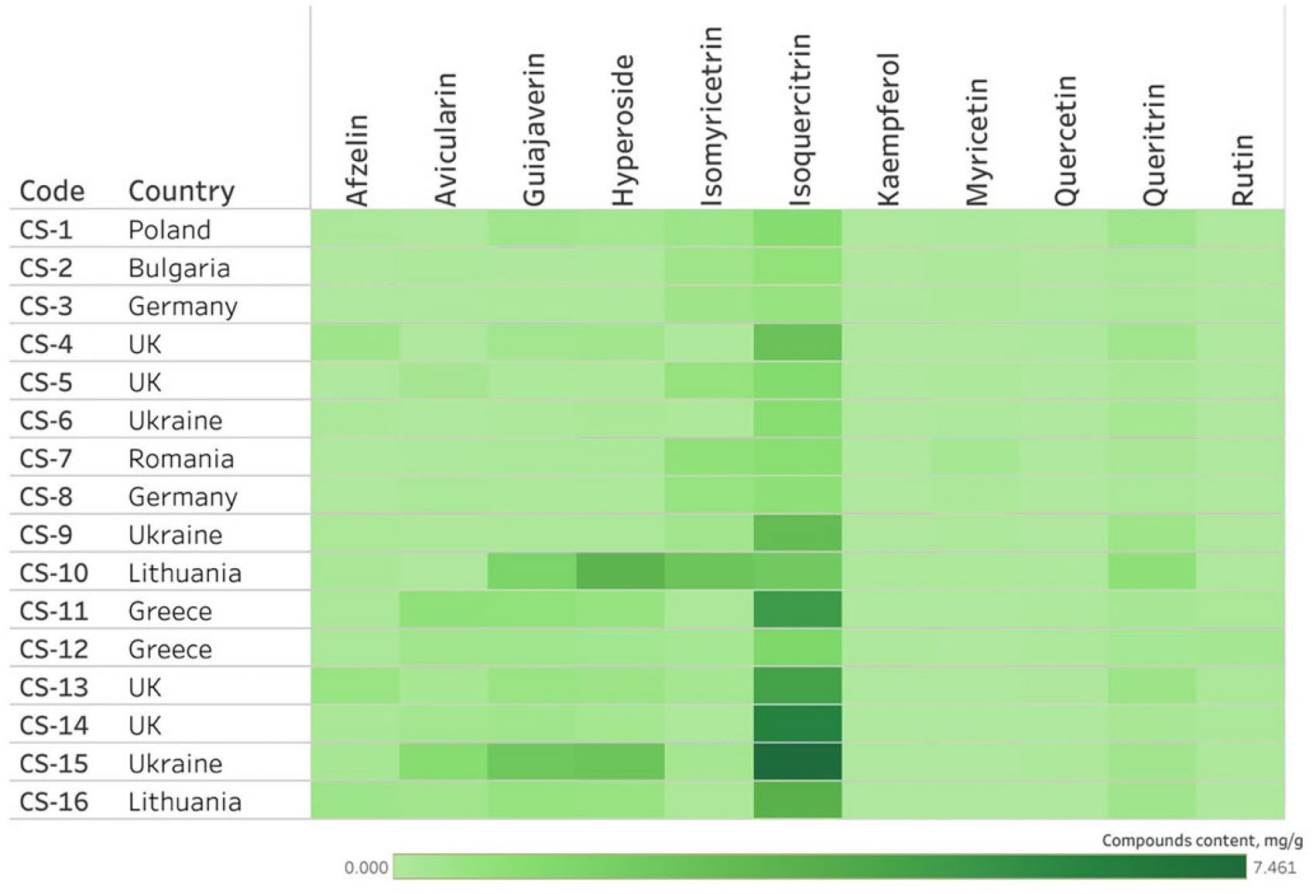
The heatmap of the flavonoid content in 16 commercial *Epilobium* samples. The color gradients show the mean concentration (in mg/g of DW) of individual flavonoids.

**Figure 6.**
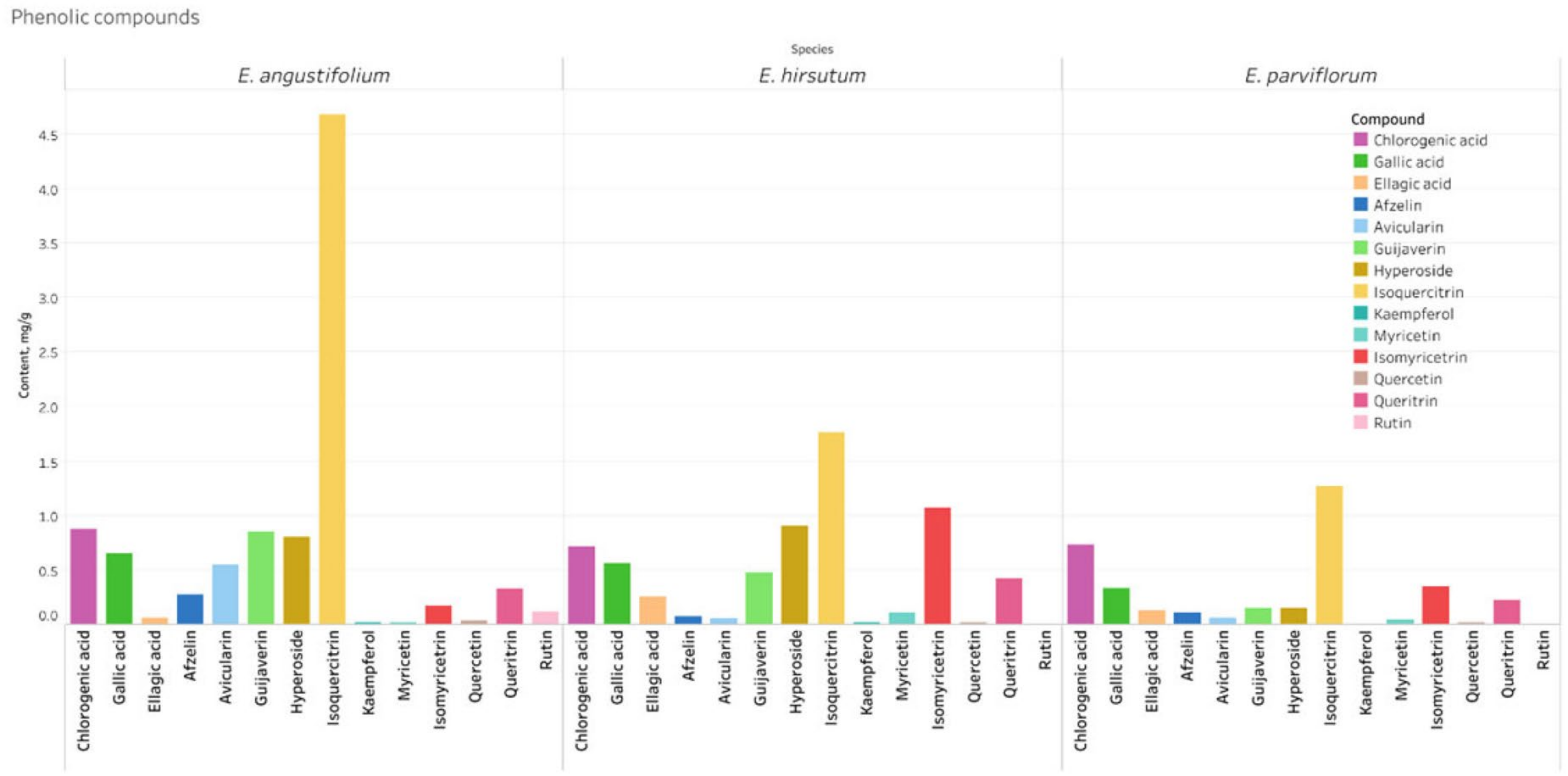
Species-dependent variation in the content of flavonoids and hydroxycinnamic acids. The bars depict the mean content (in mg/g, DW) of all phenolic compounds (excluding oenothein a and B) across three *Epilobium* species (*E. angustifolium, E. hirsutum, E. parviflorum*).

**Figure 7.**
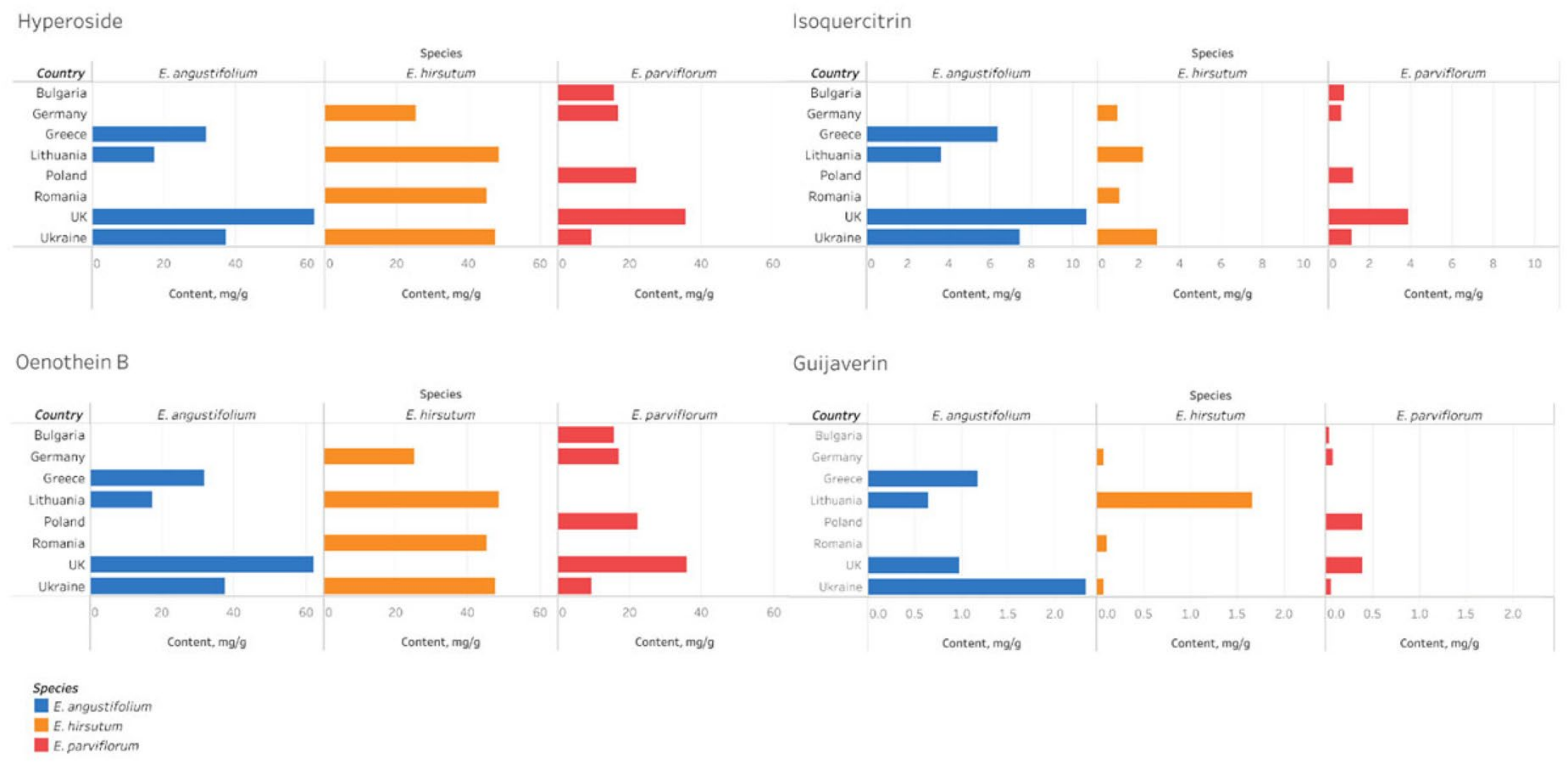
The mean content (mg/g) of four key compounds (oenothein B, hyperoside, isoquercitrin, and guaijaverin) in commercial samples of *Epilobium angustifolium, E. hirsutum* and *E. parviflorum*) from different countries.

Oenothein B exhibited significant species-specific variability (Table 2). Significant differences in mean concentrations were observed between species (Kruskal–Wallis *H* = 23.92, *p* < 0.001), with the highest levels detected in *E. hirsutum* (41.42 mg/g DW), intermediate levels in *E. angustifolium* (24.82 mg/g DW), and the lowest levels in *E. parviflorum* (16.51 mg/g DW). These differences suggest a strong relationship between species identity and ellagitannin accumulation. A similar interspecies pattern was observed for oenothein A, which was present at significantly lower concentrations in *E. parviflorum* than in *E. hirsutum* and *E. angustifolium* (Table 2).

#### Phenolic acids

The gallic acid content differed significantly between *E. parviflorum* (0.34 mg/g DW) and the other two species, with higher mean values observed in *E. hirsutum* (0.56 mg/g DW) and *E. angustifolium* (0.64 mg/g DW). Similar species-dependent variation was observed for ellagic acid, with the highest mean concentration detected in *E. hirsutum* (0.25 mg/g DW), followed by *E. parviflorum* (0.13 mg/g DW), and the lowest levels in *E. angustifolium* (0.06 mg/g DW). By contrast, no statistically significant differences were observed between species for chlorogenic acid (Table 2).

Spearman’s rank correlation analysis revealed several statistically significant associations among phenolic compounds (*n* = 16 samples). A moderate positive correlation was observed between oenothein B and gallic acid (r_s_ = 0.57, *p* < 0.001), as well as between oenothein B and ellagic acid (r_s_ = 0.40, *p* = 0.005). This suggests the coordinated accumulation of ellagitannins and related phenolic acids, indicating a relationship along the biosynthetic pathway.

#### Flavonoid glycosides and aglycones

Significant species-specific variations were observed for several flavonol glycosides. The isoquercitrin content was significantly higher in *E. angustifolium* (4.68 mg/g DW) than in *E. parviflorum* (1.27 mg/g DW) and *E. hirsutum* (1.77 mg/g DW) (Figure 5, Table 2). A similar trend was observed for guaijaverin, with mean concentration increasing from *E. parviflorum* to *E. hirsutum*, peaking in *E. angustifolium* (0.85 mg/g DW).

The mean concentration of myricetin glucoside was highest in *E. hirsutum* (1.24 mg/g DW), while *E. parviflorum* and *E. angustifolium* contained lower and comparable amounts. Several minor flavonoids, including avicularin and quercitrin, also exhibited species-specific distribution patterns (Figure 6).

Rutin was detected only in *E. angustifolium* (0.19 mg/g DW). It may be a qualitative marker of *E. angustifolium* rather than a quantitative one for distinguishing the studied species.

Overall, *E. hirsutum* was characterized by an elevated level of ellagitannins, *E. angustifolium* by a higher content of flavonol glycosides, and *E. parviflorum* by generally lower concentrations of both ellagitannins and flavonoids.

Among the flavonoids analyzed, isoquercitrin and hyperoside exhibited a strong positive correlation (r_s_ = 0.74, *p* < 0.001), indicating closely linked variation and potentially reflecting shared biosynthetic regulation. By contrast, myricetin glucoside exhibited a strong negative correlation with isoquercitrin (r_s_ = −0.63, *p* = 0.001), as well as with guaijaverin and ellagic acid (r_s_= −0.56, *p* < 0.001), suggesting the presence of competitive biosynthetic pathways. A strong positive correlation was also observed between myricetin glucoside and myricetin aglycone (r_s_ = 0.64, *p* = < 0.001). The analysis shows that commercial *Epilobium* samples are chemically diverse and cannot be considered chemically equivalent, even if they are labeled as the same species or originate from the same country.

**Figure 8.**
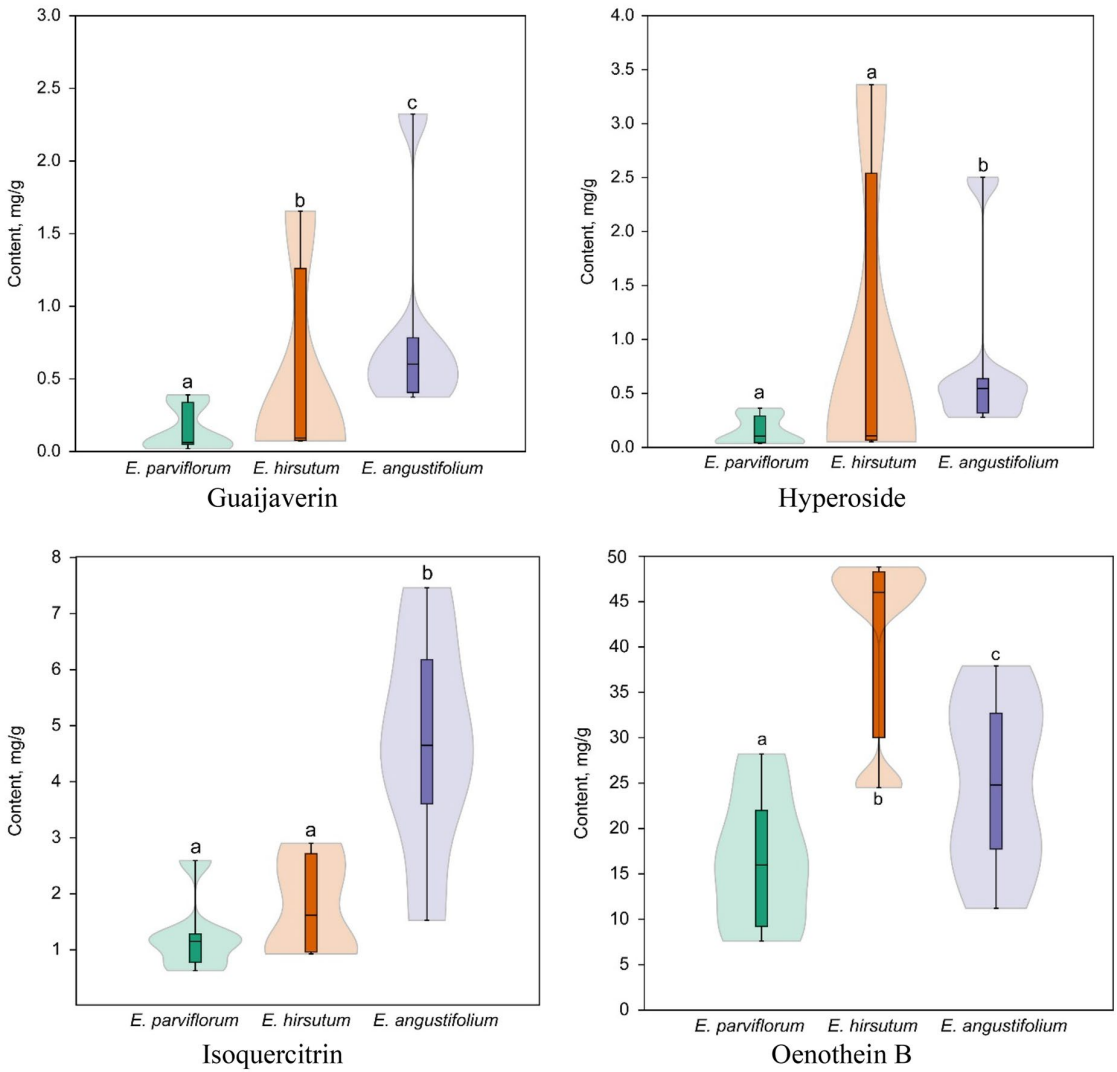
The quantity of guaijaverin, hyperoside, isoquercitrin, and oenothein B in samples of *Epilobium angustifolium*, *E. hirsutum* and *E. parviflorum*. Whiskers represent the standard deviation. Different lowercase letters at the violins denote significant differences (*p* < 0.05) between species according to the Mann-Whitney post hoc test.

**Figure 9.**
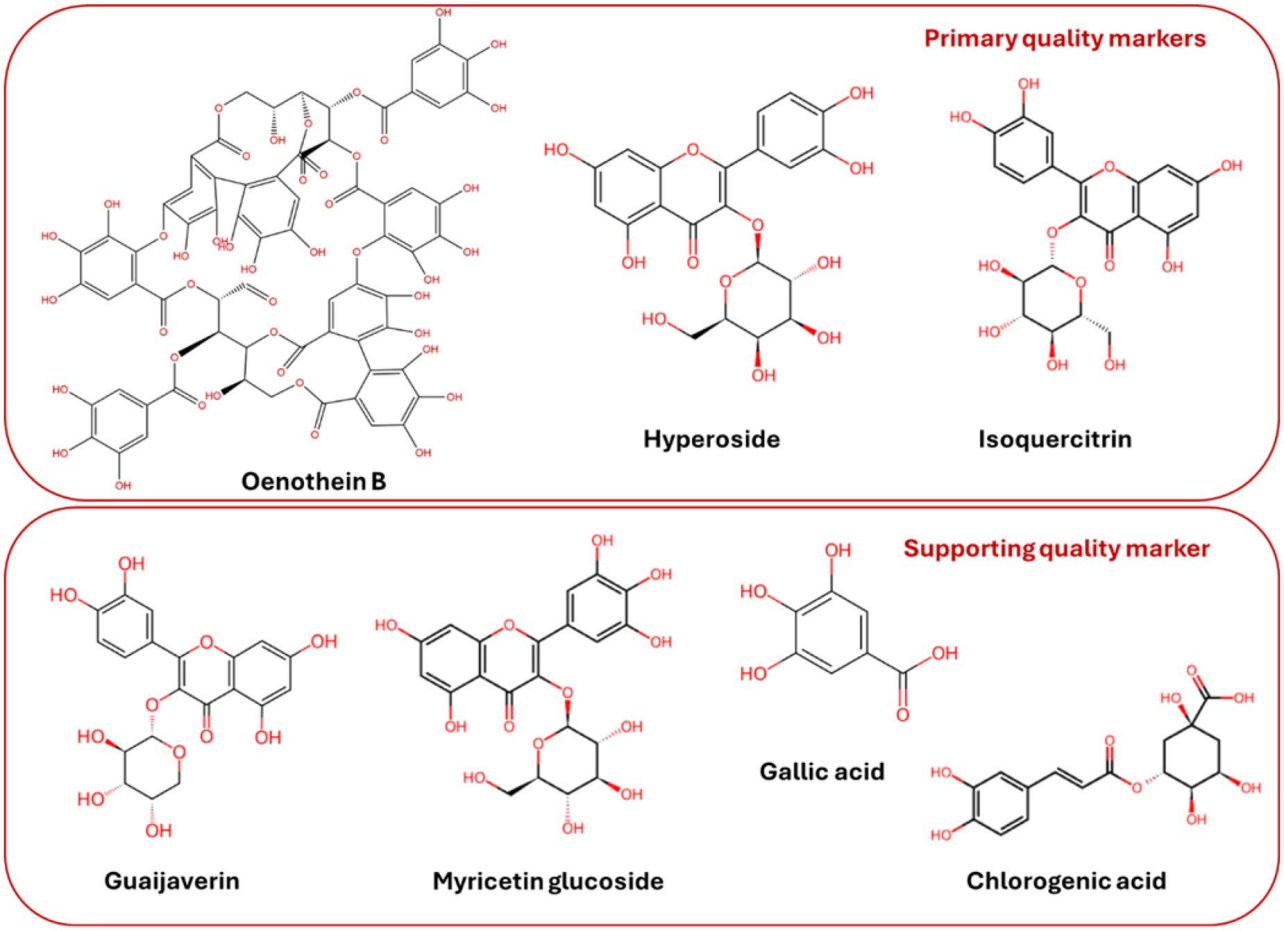
The chemical structures of the selected primary and supporting markers for the quality control of *Epilobium* samples. Chemical structures are taken from the ChemSpider database.

Clear, species-dependent differences were observed for several key phenolic compounds (Figure 7, Figure 8, Figure 9). *E. hirsutum* samples exhibited significantly higher mean concentrations of the ­ellagitannin oenothein B than commercial *E. parviflorum* and *E. angustifolium* samples. Conversely, *E. angustifolium* samples exhibited higher contents of isoquercitrin (up to 5 mg/g) and guaijaverin (up to 0.8 mg/g). However, significant intra-species variability was observed for other flavonoid glycosides (e.g., myricetin glucoside, myricetin, isoquercitrin). *E. parviflorum* samples had lower mean concentrations of oenothein B and flavonoids; some samples contained only trace amounts of hyperoside, avicularin and myricetin despite being marketed as teas of the same species. These results confirm that species identity is a significant, though not sole, determinant of chemical composition.

### Observed difference related to origin

Species-specific patterns were found to dominate over geographical origin (Figure 4 and Figure 5). It should be noted that the observed differences cannot be unambiguously attributed to geographical origin (due to limited sample size) and may reflect species-specific variability. However, variability within species by country of origin was also evident. Samples of *E. parviflorum* from the UK (CS-4 and CS-5) had an elevated content of isoquercitrin (up to 2.59 mg/g DW) and guaijaverin (0.33 mg/g DW), while the Ukrainian sample (CS-6) had a by high chlorogenic acid content (1.55 mg/g DW) and a low oenothein B concentration (9.24 mg/g DW). The Bulgarian (CS-2) and German (CS-3) samples demonstrated comparatively low flavonol and moderate ellagitannin content.

Samples of *E. hirsutum* from Romania (CS-7) and Lithuania (CS-10) showed the highest concentration of oenothein B (44.8–48.7 mg/g DW) and myricetin glucoside (0.93–2.79 mg/g DW), while the sample from Germany displayed notably lower concentration of ellagitannins (24.9 mg/g DW of oenothein B). The sample from Ukraine had increased isoquercitrin content (2.90 mg/g DW).

Samples of *E. angustifolium* exhibited the greatest internal heterogeneity. Samples from Greece (CS-11, CS-12) contained a moderate level of flavonoid glucosides, but a relatively low concentration of oenothein B (11.4–20.1 mg/g DW). By contrast, the sample from Ukraine (CS-15) was enriched in both oenothein B (37.43 mg/g DW) and hyperoside (2.50 mg/g DW). Samples from the United Kingdom (CS-13, CS-14) exhibited particularly high concentrations of isoquercitrin (4.50–6.18 mg/g DW).

Statistical analysis confirmed that geographical origin influenced the content of several compounds, including hyperoside (*H* = 31.21, *p* < 0.001), isoquercitrin (*H* = 32.52, *p* < 0.001), and guaijaverin (*H* = 25.06, *p* = 0.001). However, differences in oenothein B content across countries were not significant (*H* = 14.74, *p* = 0.064). These patterns suggest the influence of climatic and soil factors on ellagitannin content, with higher levels observed in samples from Eastern Europe (Ukraine, Lithuania, and Romania) (Figure 7). However, definitive conclusions are limited by uneven species representation and small sample sizes per country.

The results obtained demonstrate that commercial products from the genus *Epilobium* are chemically heterogeneous rather than chemically equivalent. The primary determinant of phenolic composition is species identity, particularly for ellagitannins and major flavonol glycosides. However, geographic origin introduces additional, secondary variability within species. Nevertheless, the significant variability within species indicates that species designation alone is insufficient to guarantee the chemical homogeneity of commercial products.

Due to the uneven distribution of species across countries and the small number of observations, apparent differences associated with countries are less precise than those between species. This is consistent with the heat maps (Figure 4, Figure 5, Fig. S5), in which geographic patterns are secondary to species distribution patterns.

### Selection and validation of epilobium species quality markers

The selection of potential quality markers for the chemical characterization of *Epilobium* species was based on (i) Herb MaRS-based prioritization and (ii) structural reassessment through PAINS-oriented analysis (Table 3**;** Table 4; Table S1-S2) and based on analytical suitability, abundance and discriminatory potential between samples. This two-step approach enabled us to refine the pharmacologically relevant candidates while accounting for structural features that could interfere with the analysis. Priority was given to compounds exhibiting anti-inflammatory, antiviral and antioxidant activity.

**Table 3.** Quality markers of the *Epilobium* commercial teas.

Compound	Mean (mg/g)	CV (%)	Species specificity		Relevance of the quality marker
Chlorogenic acid	0.78	45.9	Moderate		Supporting marker
Gallic acid	0.51	43.9	Moderate		Supporting marker
Ellagic acid	0.13	83.7	Low		Fingerprinting only
Oenothein A	0.06	44.5	Moderate		Fingerprinting only
Oenothein B	25.87	51.5	High		Primary marker
Avicularin	0.25	134.3	Low		Fingerprinting only
Guaijaverin	0.50	128.7	Moderate		Supporting marker
Hyperoside	0.59	160.8	High		Primary marker
Isoquercitrin	2.67	78.1	High		Primary marker
Kaempferol	0.015	81.0	Low		Fingerprinting only
Quercetin	0.026	67.6	Low		Fingerprinting only
Rutin	0.045	167.1	Moderate		Fingerprinting only
Afzelin	0.16	110.1	Low		Fingerprinting only
Quercitrin	0.31	67.6	Moderate		Fingerprinting only
Myricetin	0.05	115.8	Low		Fingerprinting only
Myricetin-glucoside	0.47	127.0	Moderate		Supporting marker
*Note***:** The species specificity and the relevance of the quality markers were assigned based on the results of the compound content and Herb MaRS ranking in Table S1.					
**Species specificity:**				**Quality marker relevance:**	
**High** – a key marker that clearly distinguishes between *Epilobium* species based on correlation and statistical analysis. **Moderate** – partially differentiates species. **Low** – marker is evenly distributed across species and is not species-specific.				**Primary marker** – Herb MaRS score is 5; it is pharmacologically and analytically important for quality control. **Supporting marker** – is useful for quantitative assessment, but secondary. **Fingerprinting only** – informative for HPLC/HPTLC-based profiling; but not critical for a quantitative quality marker.	

**Table 4.** *In Silico* PAINS and structural risk assessment of Herb MaRS candidates. Group of compounds: 1 – ellagotanins; 2 – flavonoids; 3 – phenolic acids (a – quercetin group; b – myricetin group).

Compound	Molecular formula	Molecular weight, g/mol	PAINS alerts (SwissADME or RDKit)	Redox risk	PubChem Bioassay Density
Afzelin^2a^	C_21_H_20_O_10_	432.38	0 alerts	Low	Moderate
Avicularin^2a^	C_20_H_18_O_11_	434.35	1 (catechol_A)	Moderate	High
Myricetin^2b^	C_15_H_10_O_8_	318.24	1 (catechol_A)	Elevated	High
Myricetin-3-O-glucoside^2b^	C_21_H_20_O_13_	480.38	1 (catechol_A)	Elevated	Moderate
Guaijaverin^2a^	C_20_H_18_O_11_	434.35	1 (catechol_A)	Moderate	Moderate
Quercetin^2a^	C_15_H_10_O_7_	302.24	1 (catechol_A)	Moderate	Very High
Quercitrin^2a^	C_21_H_20_O_11_	448.38	1 (catechol_A)	Moderate	High
Hyperoside^2a^	C_21_H_20_O_12_	464.38	1 (catechol_A)	Moderate	High
Isoquercitrin^2a^	C_21_H_20_O_12_	464.38	1 (catechol_A)	Moderate	High
Rutin^2a^	C_27_H_30_O_16_	610.52	1 (catechol_A)	Moderate	High
Gallic acid^2^	C_7_H_6_O_5_	170.12	1 (catechol_A)	Moderate	Moderate
Ellagic acid^2^	C_14_H_6_O_8_	302.19	1 (catechol_A)	Moderate	Moderate
Oenothein B^1^	C_68_H_48_O_44_	1568.90	1 (catechol_A); polyphenolic	Elevated	Limited
Chlorogenic acid^3^	C_16_H_18_O_9_	354.31	2 (catechol_A, Michael_acceptor)	Elevated	High

#### Primary quality markers

Oenothein B was ranked as a primary quality marker due to its high pharmacological relevance, abundance, and species-specificity within the genus *Epilobium* (Herb MaRS score of 5 out of 5). The compound has been associated with anti-inflammatory and prostate-related activities (Granica et al. 2012; Agnieszka et al. 2018a; Lasinskas et al. 2024); antiviral activity against the hepatitis C virus (HCV) (Tamura et al. 2019), and recent data have demonstrated anticoronavirus activity against the Omicron strain (71.4% at 10 μM; IC_50_ = 6.08 μM) (Uminska et al. 2025). *In vivo* studies have confirmed anti-inflammatory effects in ulcerative colitis models (Li et al. 2026), as well as angiogenic activity (Silva et al. 2024) and antitumour activity (Miyamoto et al. 1993). The clinical trial demonstrated the efficacy of *E. angustifolium* extract with a high oenothein B content in treating benign prostatic hyperplasia (Esposito et al. 2021).

The literature reports oenothein B concentration ranges from 6.1 mg/g DW to 72.9 mg/g DW. In the analyzed commercial samples, oenothein B concentration reached 48.7 mg/g DW and showed pronounced species-dependent variation. From a quality control perspective, oenothein B meets several key criteria: it is highly abundant; analytical standards are available; validated HPLC-DAD and LC-MS methods are available, and it has strong discriminatory power across species and commercial products. The substantial variability of this indicator across samples further confirms its role as an indicator of quality variability.

Hyperoside was assigned an Herb MaRS score of 5 based on its documented anti-inflammatory (Wu et al. 2007; Ku et al. 2015), antioxidant and antiviral activities. Reported antiviral effects include inhibition of hepatitis B virus (HBV) replication and secretion markers (Wu et al. 2007), as well as interaction with conserved viral protein residues in docking studies (Ahmad et al. 2016). Published concentrations of the compound in *Epilobium* species range from 0.3 to 4.6 mg/g DW. In the present study, hyperoside concentration varied widely across commercial samples (up to 2.5 mg/g DW) and consistently contributed to species discrimination, particularly in distinguishing *E. angustifolium*. Despite intra-species variability, its quantitative robustness and chemical relevance justify its designation as a primary quality marker.

Isoquercitrin was also prioritized (Herb MaRS score is 5). Although previously reported at relatively low concentrations, this study demonstrated significantly higher and analytically reliable concentrations of the compound in commercial material, particularly in *E. angustifolium* (4.5–6.2 mg/g DW). Isoquercitrin exhibited species-specificity and a strong correlation with hyperoside, reflecting coordinated flavonol biosynthesis. Its well-documented anti-inflammatory (Comalada et al. 2005) and neuroprotective (Yang et al. 2021) activities *in vivo*, as well as its antioxidant and antiviral properties (Kowalik et al. 2022; Song et al. 2025) *in vitro*, further support its biological relevance. The availability of reference standards and validated chromatographic methods confirms its suitability as a quantitative marker.

#### Supporting quality markers

Both gallic acid and chlorogenic acid have been shown to exhibit antioxidant, anti-inflammatory and antiviral properties. These include the reported inhibition of influenza virus neuraminidase and SARS-CoV-2 entry. They are also biosynthetically linked to the ellagitannin formation (You et al. 2018; Lin et al. 2022; Huang et al. 2023; Li et al. 2024). Gallic acid was consistently detected in all samples at low, yet quantifiable concentration (0.3–0.6 mg/g DW), showing a moderate correlation with oenothein B.

However, their widespread occurrence across medicinal plants, limited species specificity, and weaker discriminatory power between *Epilobium* species reduce their suitability as primary quality markers under the Herb MaRS criteria. Similar limitations were observed for guaijaverin and myricetin-glucoside (Herb MaRS score ≤ 3), which exhibited low-to-moderate concentrations and high intra- and interspecific variability. While these compounds remain valuable for chromatographic fingerprinting and correlation analysis of overall chemical variability, they are less robust as primary markers.

An analytical analysis shows that oenothein B, hyperoside, and isoquircitrin fulfill the central Herb MaRS criteria:

- pharmacological relevance consistent with traditional and experimental data;
- sufficient concentration in plant material for reliable quantification;
- availability of reference standards;
- compatibility with routine HPLC-based quality control workflows.

Importantly, these compounds also exhibited high coefficients of variation across commercial products, indicating their dual relevance as both biologically significant compounds and as indicators of quality variability.

#### Structural assessment and PAINS analysis

PAINS analysis revealed that most of the major *Epilobium* polyphenols, particularly the flavonoids, triggered structural alerts due to the presence of catechol and pyrogallol moieties (Table 4). These substructures are associated with redox activity, metal chelation and nonspecific protein interactions, all of which may lead to false-positive results in certain *in vitro* assays (Baell and Holloway 2010).

Ellagitannins, such as oenothein B, generated multiple alerts related to catechol or pyrogallol and were classified as highly polyphenolic structures with potentially aggregation-prone properties (Raya-Morquecho et al. 2025). Similarly, flavonoid glycosides triggered redox-related filters.

However, none of the compounds contained high-risk synthetic PAINS scaffolds (e.g., rhodanines, berberine, beta-sitosterol, azo dyes or reactive enedione systems). The alerts observed here primarily relate to the redox features of plant polyphenols rather than to artefactual assay-interference moieties (Baell, 2014).

Crucially, oenothein B, isoquercetin, and hyperoside were selected as quality markers based on chemical specificity, quantitative reproducibility and demonstrated *in vivo* relevance, rather than drug-likeness assumptions. Published *in vivo* studies support their biological activity (Table S2), mitigating concerns that their prioritization is solely due to assay artifacts.

Thus, the PAINS screening did not automatically eliminate candidates; rather, it enabled a critical reassessment of their structure. This step clarified that signals associated with redox processes, which are common in plant polyphenols, do not preclude the usefulness of these compounds as quality markers when interpreted in the context of chemically relevant quantitative stability and *in vivo* confirmation data.

It is important to note that the frequent presence of redox-active catechol and pyrogallol moieties suggests that some of the claimed anti-inflammatory and antiviral activity of *Epilobium* polyphenols may reflect assay-specific enhancement rather than specific pharmacological effects. Therefore, pharmacological evaluation should not be equated with clinical data.

## Discussion

### Chemical heterogeneity among commercial Epilobium tea products

The present results show the significant chemical heterogeneity among commercial *Epilobium* tea samples, as demonstrated by HPLC and HPTLC analyses. The quantitative data revealed significant variability in the content of main phenolic compounds across the products, even among those labeled as the same species. The main compounds, such as oenothein B, hyperoside, and isoquercitrin, exhibited high coefficients of variation (40–160%; Table 3).

Although statistically significant differences were observed between species, considerable overlap in concentration ranges was evident. The heat map (Figure 4, Figure 5, Fig. S4) revealed marked differences in the relative abundance of phenolic compounds, such as oenothein B, hyperoside, and isoquercitrin, among products from different countries, as reflected by color intensity (e.g., Fig. S4 for tannins). Samples from countries such as the United Kingdom and Ukraine exhibited similar chemical characteristics, indicating that geographic origin alone is not a consistent determinant of phenolic profile.

This suggests that the designation of a species alone is insufficient to account for the quantitative variability in bioactive metabolites among commercial products. Geographic origin was found to further modulate phenolic profiles (Fig. S7), yet no consistent origin-specific pattern was evident. Instead, the observed heterogeneity is likely due to multiple interacting factors, such as harvest time, plant part, environmental stress, post-harvest processing, drying conditions and storage.

Several other factors could not be accounted for, yet they strongly influence the accumulation of specialized metabolites in *Epilobium* species. These include:

- The exact site where the starting plant material was collected, including altitude, soil type, and microclimatic conditions.
- Time of harvest and phenological stage, as ellagitannins and flavonoids are known to vary across the vegetative and flowering periods.
- Length of the vegetation period and exposure to environmental stress during plant development.
- Plant part used (e.g. leaves, aerial parts, flowering tops), as these may differ substantially in their polyphenolic profiles.
- Harvesting practices, including manual versus mechanical collection and cutting height.
- Post-harvest processing, particularly fermentation or enzymatic treatment, can be applied to some *E. angustifolium* teas. This study did not analyze fermented tea.
- Drying method and temperature, as these critically affect the stability of ellagitannins and flavonoid glycosides.
- Storage conditions, including duration, humidity, light exposure, and oxygen availability.
- Transportation and distribution chains, which may further contribute to the degradation or transformation of sensitive compounds.

These factors are known to modulate the biosynthesis, degradation, and interconversion of polyphenols and ellagitannins. This directly influences the qualitative and quantitative profiles of *Epilobium* commercial products.

Our findings regarding the variability of polyphenols in commercial *Epilobium* teas are consistent with and build upon previous research on wild and cultivated populations of *Epilobium* species. For example, the wide range of oenothein B concentration observed in our samples (7.9–48.7 mg/g DW, with mean values of 16.5 ± 7.6 mg/g DW for *E. parviflorum*, 41.4 ± 11.1 mg/g DW for *E. hirsutum*, and 25.1 ± 10.0 mg/g DW for *E. angustifolium*) is consistent with reports from wild *E. angustifolium* populations. Baert et al. (2017) documented variability in ellagitannins, including oenothein B, among populations of *E. angustifolium,* attributing it to genetic and environmental factors, such as soil composition and climate. Similarly, Monschein et al. (2015) found that the phenolic content of wild *E. angustifolium*, including flavonoids such as hyperoside and isoquercitrin, varied significantly with altitude, with higher elevations correlating with higher ellagitannin content. This reflects the trend observed in our samples from Eastern Europe (e.g., Ukraine and Lithuania, up to 48.7 mg/g DW), which often contain higher levels of oenothein B (up to 48.7 mg/g DW) and may indicate cooler climates or growing conditions compared to samples from Western Europe (e.g., the UK and Germany), which often contain less than 25 mg/g DW. By contrast, our commercial samples exhibited greater intraspecific heterogeneity (CV >50% for oenothein B) than the more controlled wild collections examined in these studies. This is likely due to additional factors, such as post-harvest processing and fermentation, as observed in our *E. angustifolium* teas. This is supported by Maruška et al. (2014), who tracked fluctuations in flavonoids during the vegetative stages of *E. angustifolium* and reported a hyperoside concentration (0.1–1.2 mg/g DW) comparable to our mean value (0.80 ± 0.91 mg/g DW), but with less variability. This suggests that commercial processing amplifies inconsistencies. Furthermore, Dreger et al., 2021 highlighted genotypic differences in polyphenolic content among *E. angustifolium* cultivars, with isoquercitrin content ranging from 1 to 6 mg/g DW. This aligns closely with our findings of elevated content in *E. angustifolium* (up to 7.5 mg/g DW in samples from the UK and Ukraine) and supports the idea that species-specific patterns are stronger than geographical ones. However, unlike (Tóth et al., 2006), who reported stable phenolic profiles in commercial *E. parviflorum* extracts analyzed by LC-MS/MS (e.g., myricetin, quercetin, and kaempferol glycosides dominant, and high antioxidant capacity EC_50_ = 1.71 ± 0.05 μg/mL), our broader multi-species analysis of herbal teas revealed greater variability in phenolic acids (CV >40%), emphasizing the need for standardization by species and origin to mitigate nonequivalence in marketed products.

The data on the quality and quantitative content of phenolic compounds in our commercial *Epilobium* samples are consistent with those from our previous study of *E. angustifolium* samples collected in Ukraine during three flowering phases (Ivanauskas et al. 2024).

Compared with studies on wild or cultivated *Epilobium* populations, the commercial samples displayed significantly greater intraspecific variability. This suggests that post-harvest handling, blending practices and supply chain factors can increase chemical heterogeneity beyond natural environmental variations. Therefore, it cannot be assumed that commercial products are chemically or therapeutically interchangeable.

### Implications for the selection of quality markers

Rational selection of quality markers is critical when considering pronounced variability. The Herb MaRS framework offers a structured way to prioritize candidate markers based on their pharmacological relevance, abundance, traceability, analytical feasibility, and safety considerations. Using this system, oenothein B, hyperoside, and isoquercitrin were identified as primary quality markers (Figure 8; Table S2).

The focus on compounds with prostate-related, anti-inflammatory, and antioxidant properties reflects the main traditional and pharmacological applications of *Epilobium* species (Esposito et al. 2021; Stolarczyk M, Naruszewicz M, et al. 2013). While some compounds, such as oenothein B, hyperoside, and gallic acid, have been reported to exhibit additional properties (e.g., antiviral activity), the current selection emphasizes the relevant markers in the context of reported traditional use and literature-documented biological activity. This provides a robust basis for the quality assessment of commercial herbal teas and extracts.

Importantly, these compounds fulfill the criteria of Herb MaRS and represent the most quantitatively variable constituents across commercial products. This variability enhances their value as indicators of quality inconsistency, linking the selection of markers directly to the assessment of product heterogeneity. Therefore, marker prioritization and variability assessment are interdependent processes rather than separate analytical exercises. Although supporting compounds such as gallic acid, chlorogenic acid, guaijaverin, and myricetin derivatives contributed to chemometric discrimination, they lacked the necessary specificity and robustness to serve as primary quality markers. The distinction between primary, supporting and fingerprinting markers highlights the need for a multi-level quality assessment strategy. Given the variability in commercial *Epilobium* material and the availability of pharmacological data, a new approach to testing raw material quality and developing a pharmacopeial monograph is required.

### Structural reassessment and methodological implications

A key methodological insight of this study concerns the need for a more systematic assessment of the validity of pharmacological data that underpin the development of chemical analytical protocols. Several major polyphenolic constituents identified in *Epilobium* species activate structural alerts in the PAINS filter (Table 4), primarily due to the presence of catechol and pyrogallol groups associated with redox activity and potential assay interference (Baell and Holloway 2010; Magalhães et al. 2021).

Such alerts do not automatically render these compounds invalid as quality markers. However, they highlight a potential issue: prioritizing compounds for pharmacological research based solely on *in vitro* bioactivity may be influenced by nonspecific, redox-driven assay effects. This is particularly relevant for polyphenol-rich plant preparations, for which frequent-hitter behavior is well documented (Newman, 2021; Gilberg et al., 2017).

In the present study, the selection of oenothein B, hyperoside, and isoquercitrin (Figure 9) was not based on assumptions of drug-likeness, but rather on chemical specificity, quantitative robustness, analytical reproducibility, and supporting *in vivo* evidence (Miyamoto et al. 1993). Therefore, PAINS screening was not applied as an exclusion filter for compounds (Magalhães et al. 2021), but rather as a tool for contextual interpretation of reported bioactivities. This enabled a critical evaluation of whether the selected markers reflected biological relevance or were influenced by assay-related artifacts.

The presence of one or more catechol or pyrogallol groups should be considered when interpreting the results, as these compounds may have limited specificity for interpreting pharmacological activity and therapeutic relevance (Baell and Holloway 2010). However, they are species-specific quality markers (specific to *Epilobium*) and chemically relevant for the genus.

Flavonoids, such as quercetin derivatives (e.g., hyperoside, isoquercitrin) and myricetin derivatives, consistently trigger catechol-related alerts and α,β-unsaturated carbonyl (Jackson et al., 2017). Their broad *in vitro* activity profiles and high bioassay density are consistent with the “frequent hitter” behavior known for redox-active flavonoids (Fushimi, 2026).

In addition to catechol-related alerts, there is a Michael acceptor alert for phenolic acids (Dinkova-Kostova, 2007). Moreover, the high frequency of positive *in vitro* results for anti-inflammatory and antioxidant activity, due to redox-active structural features, may contribute to nonspecific reactivity, which should be considered when interpreting reported bioactivities in the context of selective pharmacological mechanisms. This is based on the interpretation of pharmacological data in the literature, while the study’s main results are derived from chemical profiling and marker selection. Due to their limited genus specificity, such compounds can only be considered supporting chemical markers for analysis rather than bioactive metabolites.

**Figure 10.**
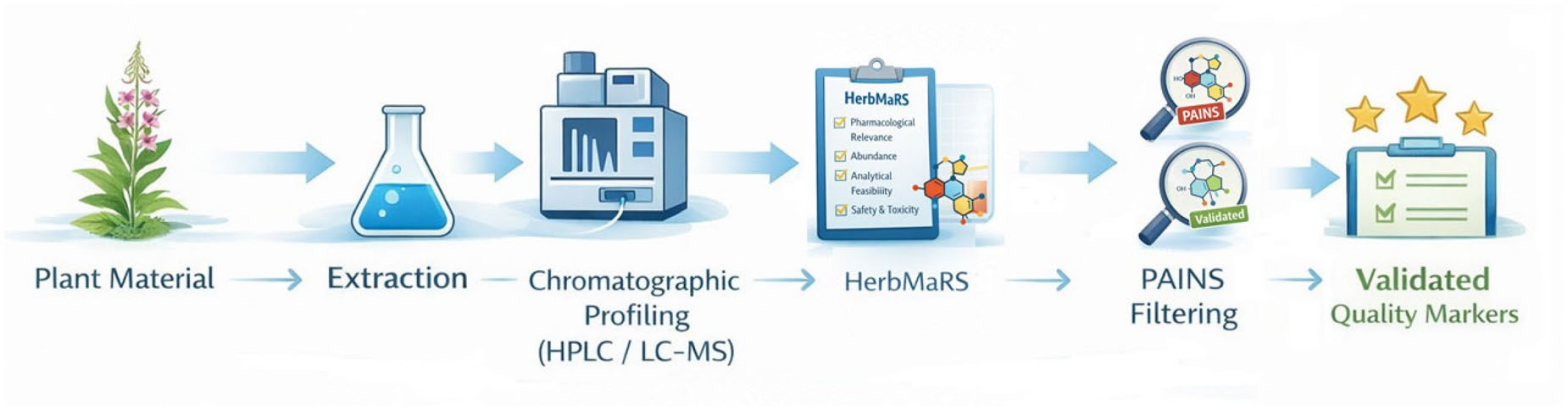
Workflow for the selection of validated quality markers from plant material. Figures were generated using the DALL·E model (OpenAI) based on detailed author-defined prompts and subsequently selected and curated by the authors.

This two-step approach involves prioritization based on the Herb MaRS (Bensoussan et al. 2015), followed by reevaluation of structural deficiencies, thereby providing a more robust basis for selecting markers in polyphenol-rich herbal products (Figure 10).

### Broader implications of chemical definition and standardizing Epilobium products

The combined evidence from quantitative variability, multivariate analysis, and marker prioritization highlights the need for species- and origin-specific quality control strategies. There is no chemical data to support uniform recommendations for dosage or therapeutic equivalence across products labeled as *Epilobium*.

The integration of targeted quantitative markers with chromatographic fingerprinting and statistical modeling provides a practical way to improve chemical characterization. Chromatographic fingerprinting, with appropriate quantification of marker compounds, aligns with the best-practice recommendations for phytopharmaceutical research set out in the ConPhyMP guidelines (Heinrich et al. 2022).

Future studies could combine metabolomic profiling with genetic authentication to better understand the influences of taxonomy, the environment and processing. While the present study is limited by its small sample size and reliance on labeled species, it demonstrates substantial, analytically measurable chemical heterogeneity among commercial *Epilobium* products.

From a regulatory and pharmacopeial perspective, the observed chemical diversity among commercially available *Epilobium* products indicates that any future development of a Pharmacopeia monograph will require clearly defined species identity for raw materials and a strategy for quantification/standardization based on multiple markers. Given the high intra- and interspecies variability demonstrated here, the development of such a monograph should be based on robust chemical datasets and a critical assessment of pharmacological evidence, rather than on traditional usage alone.

## Conclusions

This study shows that the chemical composition of commercial *Epilobium* products is mainly determined by the species used. While species identity is a key factor in composition, significant intra-species variation and overlapping concentration ranges suggest that products sold under the same plant name are not chemically equivalent. Geographic origin may influence the phenolic profiles. However, these effects cannot be clearly separated from species-related variation, and species designation alone is insufficient to ensure compositional consistency. Oenothein B, hyperoside, and isoquercitrin were identified as the most informative quality markers, as they combine chemical specificity, quantitative robustness, analytical feasibility and potential biological relevance. Notably, these compounds were also among the most variable constituents across commercial Epilobium products, highlighting their dual significance as both chemically informative markers and indicators of compositional variability.

This work highlights broader methodological implications. Although the Herb MaRS framework offers a logical approach to prioritizing candidate markers, our findings show that “pharmacological” ranking (based on uncritically used pharmacological data) alone is inadequate for developing analytical protocols, with polyphenol-rich, berberine or beta-sitosterol plants being of particular concern. Structural reassessment using PAINS-oriented analysis is a necessary second-stage assessment step to ensure that the selected markers reflect constituents that are both chemically robust and reliably interpretable in a pharmacological context, rather than potential assay artifacts (Heinrich et al. 2022). Integrating structural liability assessment strengthens the reliability of marker confirmation and improves the scientific validity of quality control strategies.

Overall, this work emphasizes the need to move away from generic labeling and single-marker approaches, and toward a more comprehensive, evidence-based assessment and a structurally informed framework for quantified/standardized herbal products. The findings of this study are based on phytochemical analysis of commercial products, while the pharmacological aspects are derived from the literature and should be interpreted with caution. Such improvements are necessary to enhance the reproducibility, transparency and traceability of the rapidly expanding herbal tea and dietary supplement market. They also have broader implications for the development of methodologies to determine quality markers in phytopharmaceutical research.

## Supplementary Material

Supplementary material_v2.pdf

## Data Availability

All the data on which this study is based are presented in the article.
