# Supplementary material for: The effect of provenance and species on the chemical composition of Epilobium herbal tea
Source: Pharm Biol. 2026 May 18;64(1):783–806. doi: 10.1080/13880209.2026.2672676 (PMC13185070; doi:10.1080/13880209.2026.2672676)
Supplement: Supplementary material_v2.pdf [file IPHB_A_2672676_SM6424.pdf]

Supplementary materials

The effect of provenance and species on the chemical composition of *Epilobium* herbal tea

Olha Mykhailenko[1,2*], Banaz Jalil[1], Kateryna Uminska[3], Liudas Ivanauskas[4], Zigmantas Gudžinskas[5], Michael Heinrich [1,6]

[1]Pharmacognosy and Phytotherapy Group, UCL School of Pharmacy, London, United Kingdom o.mykhailenko@ucl.ac.uk (O.M.*); b.jalil@ucl.ac.uk (B.J.); m.heinrich@ucl.ac.uk (M.H.); [2] National University of Pharmacy, Kharkiv, Ukraine; [3]Zhytomyr Basic Pharmaceutical Professional College, Zhytomyr, Ukraine, uminska.kateryna@pharm.zt.ua; [4] Department of Analytical and Toxicological Chemistry, Lithuanian University of Health Sciences, Kaunas, Lithuania, Liudas.Ivanauskas@lsmu.lt; [5] State Scientific Research Institute Nature Research Centre, Laboratory of Flora and Geobotany, Vilnius, Lithuania zigmantas.gudzinskas@gamtc.lt; [6]Department of Pharmaceutical Sciences and Chinese Medicine Resources, Chinese Medicine Research Center, College of Chinese Medicine, China Medical University, Taichung, Taiwan.

* Correspondence: o.mykhailenko@ucl.ac.uk

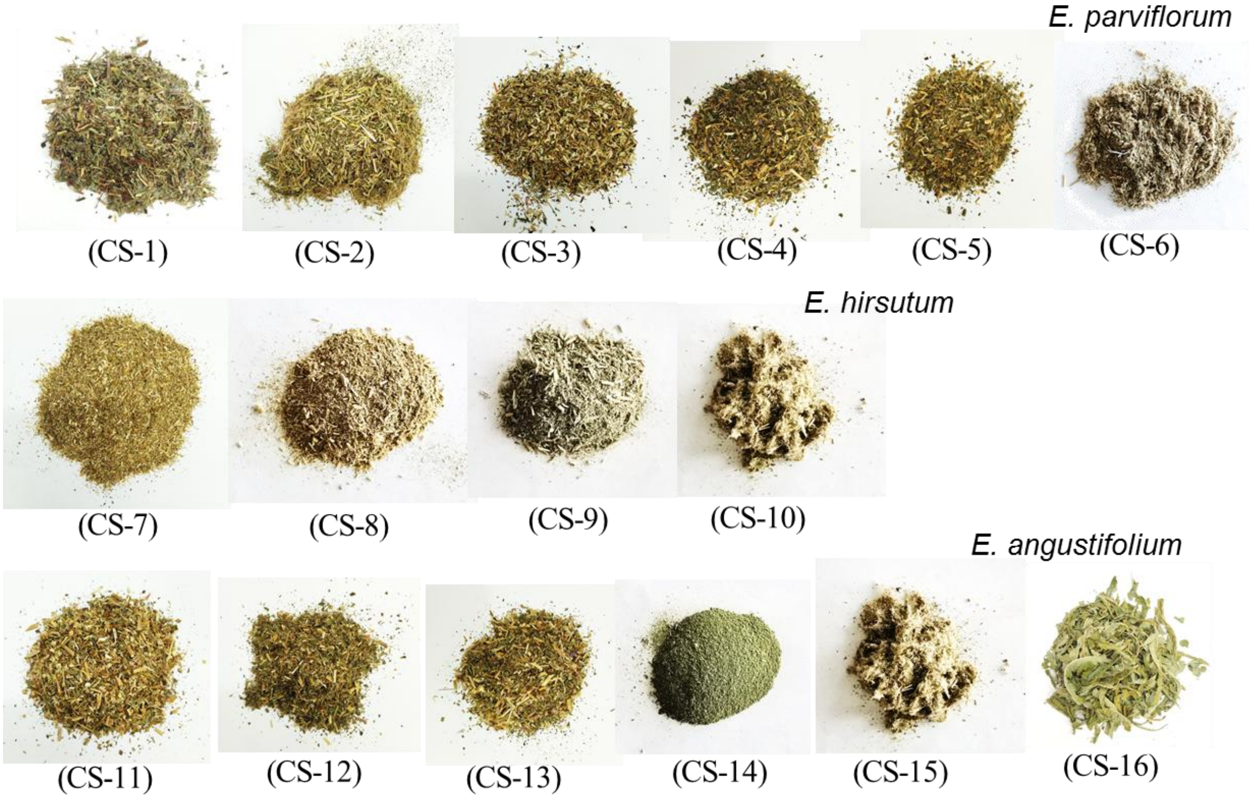

Figure S1. The appearance of the commercial *Epilobium* tea samples. Grinding by the manufacturer.

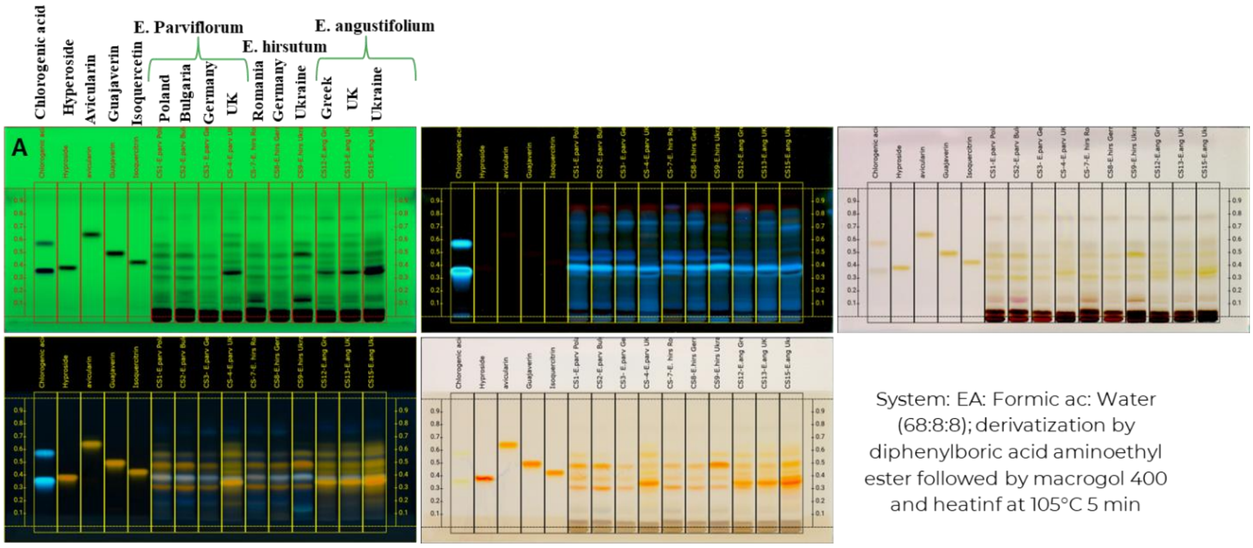

Figure S2. HPTLC profiles under UV 254 nm (A), UV 366 nm (B) and white light (C) prior to derivatisation, and under UV 366 nm after derivatisation (D), and white light after derivatisation (E).

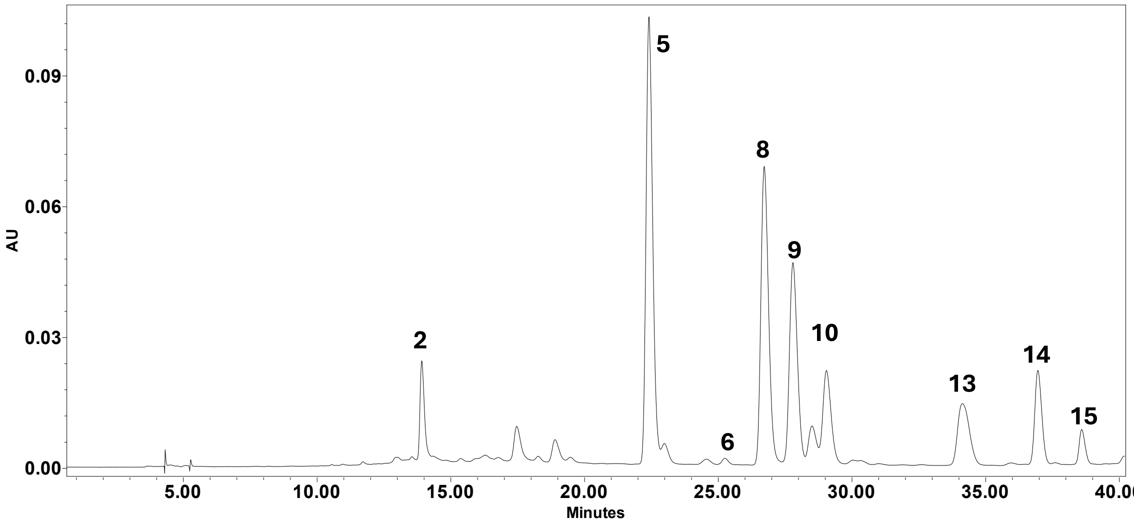

A

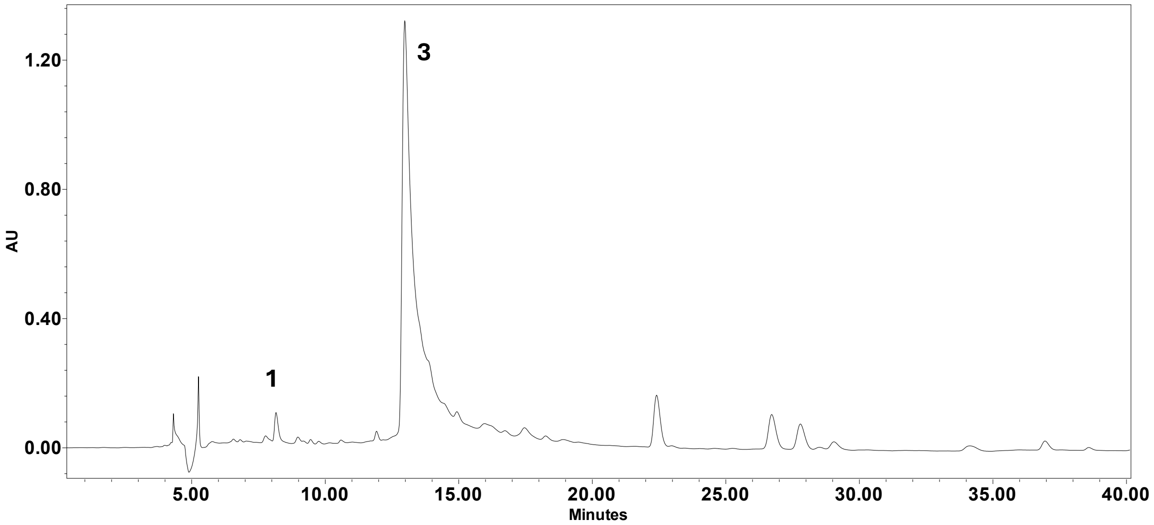

B

Figure S3. HPLC-DAD chromatograms of the methanol (50%) extracts of *Epilobium hirsutum* (CS10) samples. The chromatograms were recorded at 350 nm (A) for the identified polyphenols and at 219 nm (B) for the tannins. Peaks: 1 – gallic acid; 2 – chlorogenic acid; 3 – oenothein B; 4 – oenothein A; 5 – myricetin glucoside; 6 – ellagic acid; 7 – rutin; 8 – hyperoside; 9 – isoquercitrin; 10 – guaijaverin; 11 – avicularin; 12 – quercitrin; 13 – myricetin; 14 – afzelin; 15 – kaempferol; 16 – quercetin.

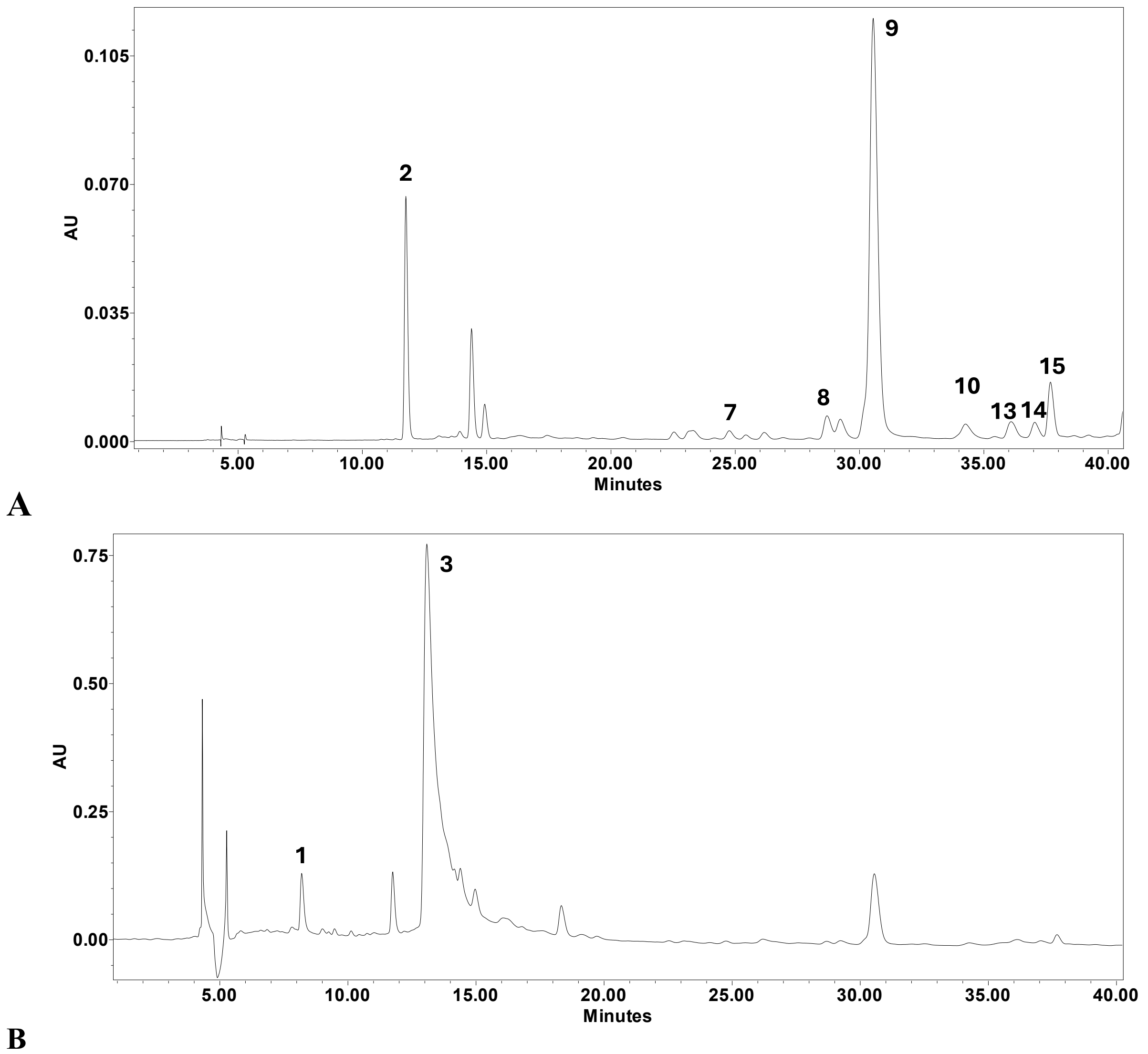

Figure S4. HPLC-DAD chromatograms of the methanol (50%) extracts of *Epilobium angustifolium* (CS14) samples. The chromatograms were recorded at 350 nm (A) for the identified polyphenols and at 219 nm (B) for the tannins. Peaks: 1 – gallic acid; 2 – chlorogenic acid; 3 – oenothein B; 4 – oenothein A; 5 – myricetin glucoside; 6 – ellagic acid; 7 – rutin; 8 – hyperoside; 9 – isoquercitrin; 10 – guaijaverin; 11 – avicularin; 12 – quercitrin; 13 – myricetin; 14 – afzelin; 15 – kaempferol; 16 – quercetin.

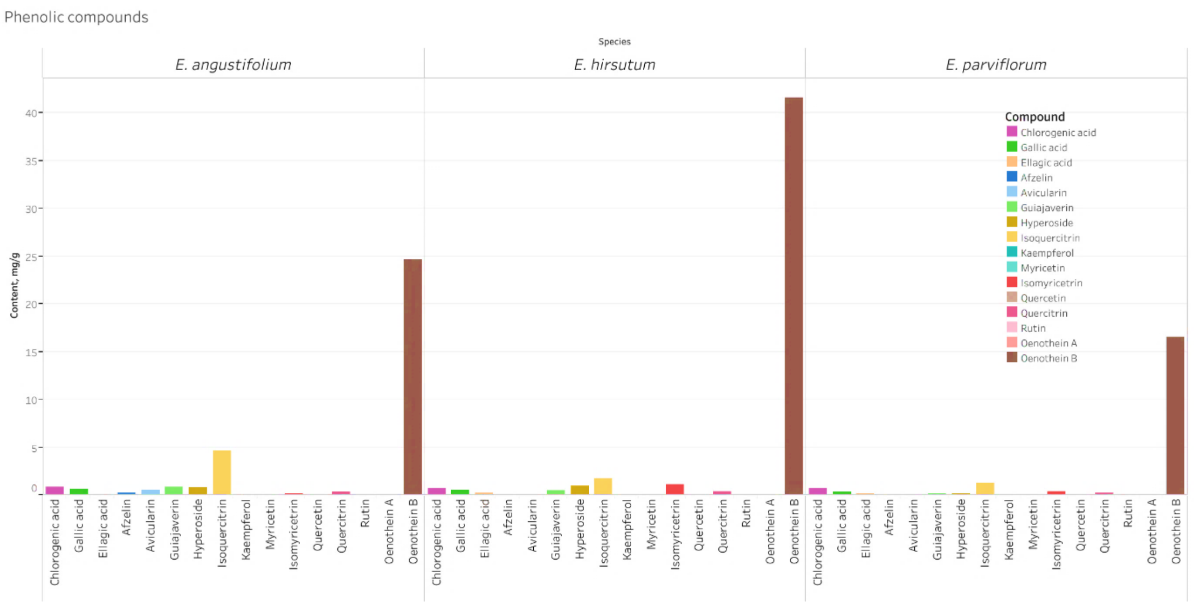

Figure S5. Species-dependent variation in phenolic compounds. The bars depict the mean content (mg/g DW) of key compounds (e.g., chlorogenic acid, gallic acid, oenothein B) in three *Epilobium* species (*E. angustifolium, E. hirsutum, E. parviflorum*).

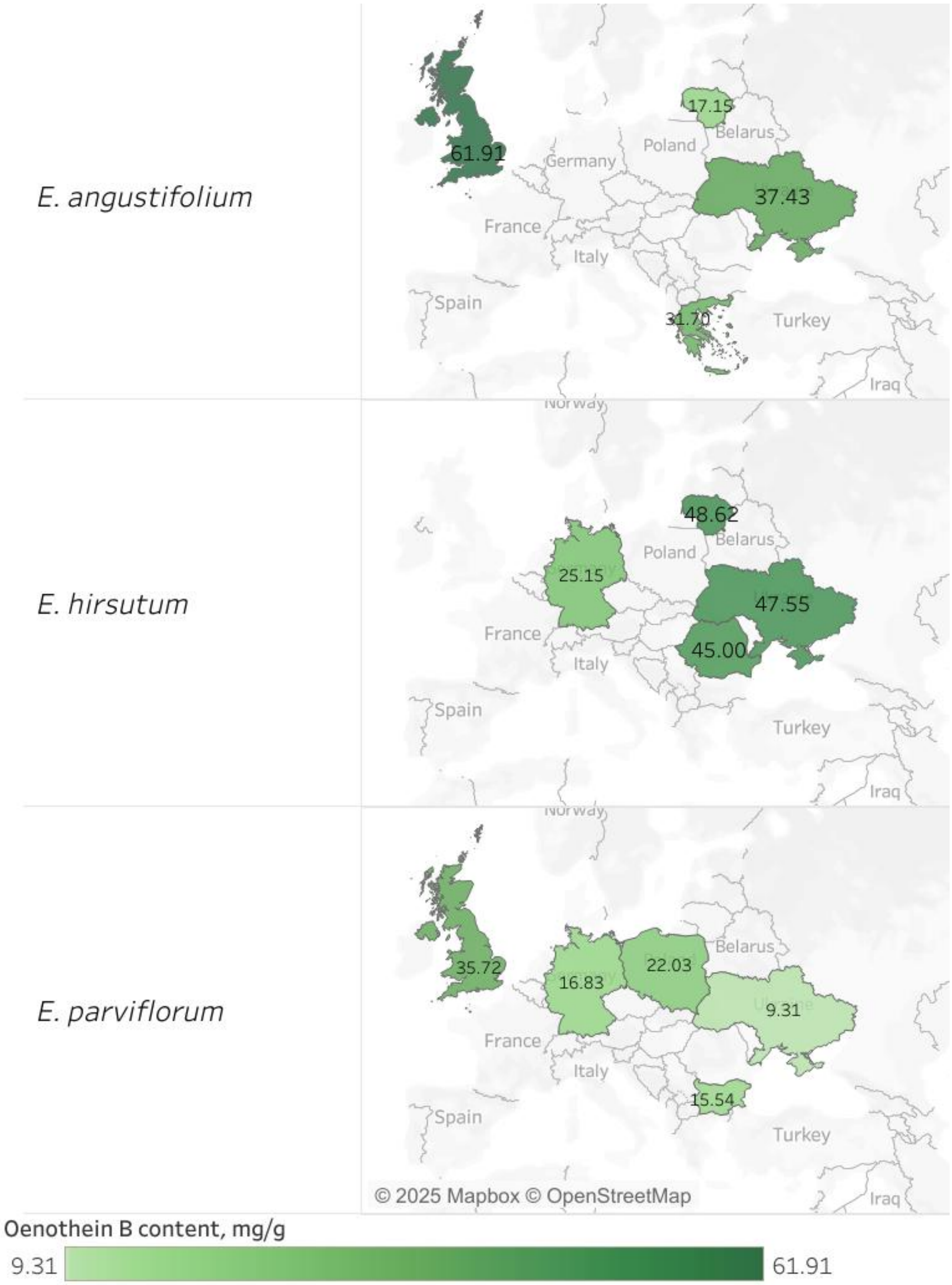

Figure S6. The distribution of oenothein B across *Epilobium* species and by country of origin. The colour intensity corresponds to the mean concentration of oenothein B (mg/g DW), revealing geographic and species-specific patterns of this key ellagitannin.

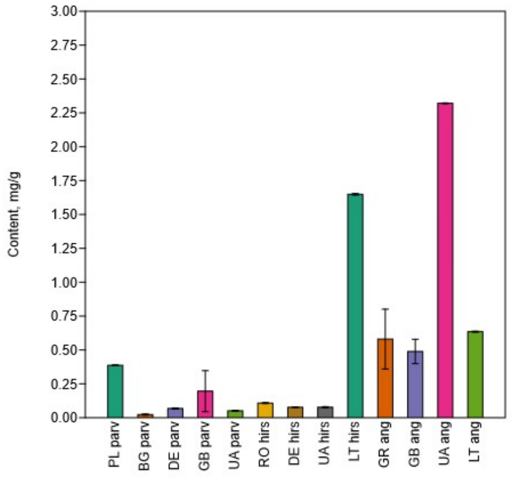

Guaijaverin (a)

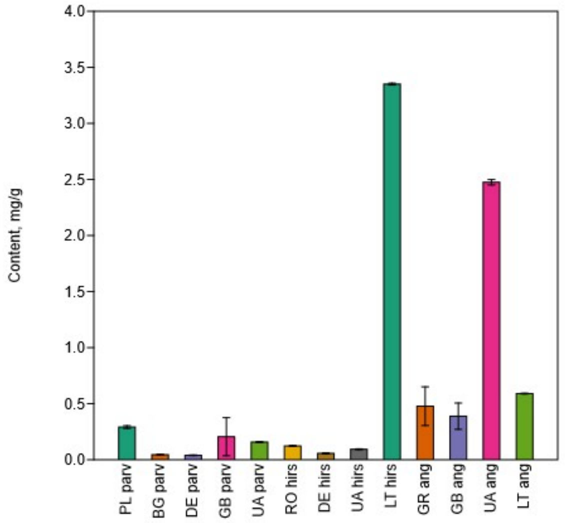

Hyperoside (b)

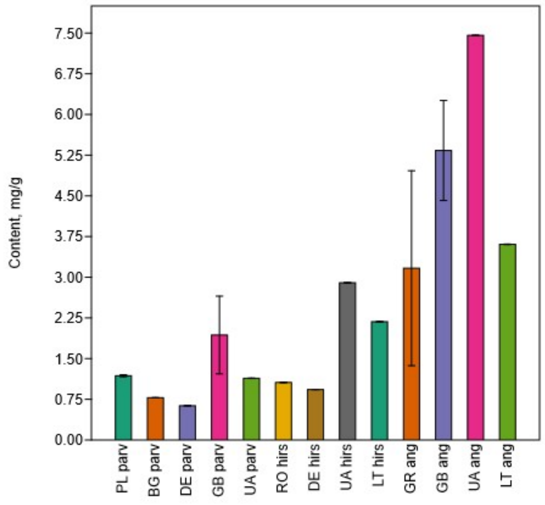

Isoquercitrin (c)

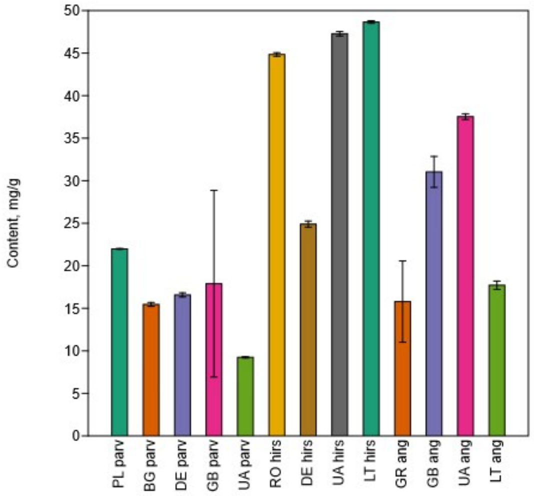

Oenothein B (d)

Figure S7. The quantity of guaijaverin, hyperoside, isoquercitrin and oenothein B in *Epilobium* samples originating from different countries.

Table S1. Specificity of 11 quantified compounds

**Phenolic acids**			**Tannins**		
Chlorogeic acid: 11.95 min. λmax: 218; 326 nm	Ellagic acid: 22.48 min. λmax: 253; 354 nm	Gallic acid: 6.10 min. λmax: 216; 271 nm	Oenothein A: 12.94 min. λmax: 217; 264 nm	Oenothein B: 10.66 min. λmax: 217; 364 nm	
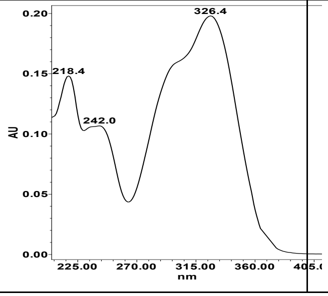	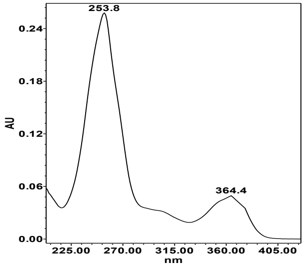	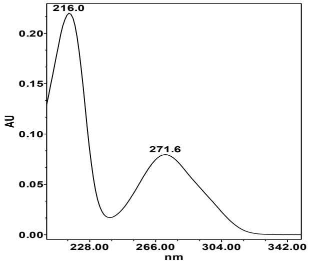	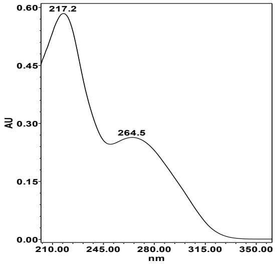	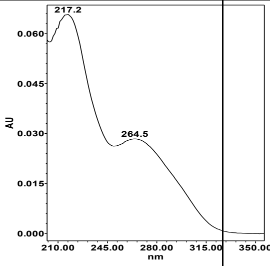	
**Flavonoids**					
Avicularin (=Quercetin 3-*α*-L-arabinofuranoside): 33.55 min. λmax: 266, 327 nm	Guajaverin (=Quercetin 3-O-*α*-L arabinopyranoside): 28.31 min. λmax: 256; 356 nm	Hyperoside (= Quercetin 3-O-*β*-D-galactopyranoside): 23.62 min. λmax: 256; 353 nm	Isoquercetrin (=Quercetin 3-O-*β*-D-glucopyranoside): 24.50 min. λmax: 256; 353 nm	Isomyricitrin (=Myricetin 3-O-*β*-D-glucopyranoside): 18.51 min. λmax: 267; 357 nm	Myricetin (3 3′ 4′ 5 5′ 7-hexahydroxyflavone): 35.60 min. λmax: 253; 376 nm
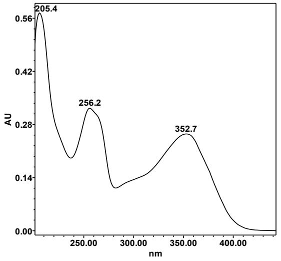	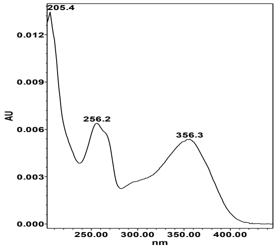	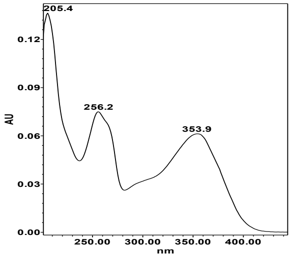	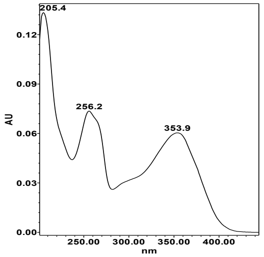	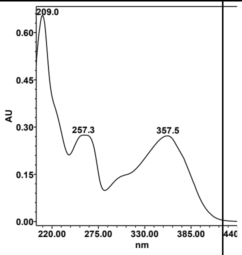	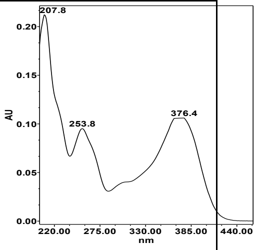

Afzelin (=Kaempferol-3-rhamnoside): 37.95 min. λmax: 264, 343 nm	Quercetin: 43.80 min; λmax: 255, 364 nm	Quercitrin (=Quercetin 3-rhamnoside), 31.07 min; λmax: 256, 347 nm	Rutin (=Quercetin 3-rutinoside), 22.52 min; λmax: 256, 353 nm		
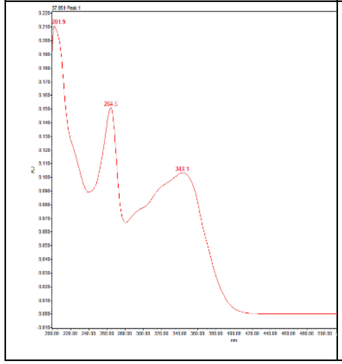	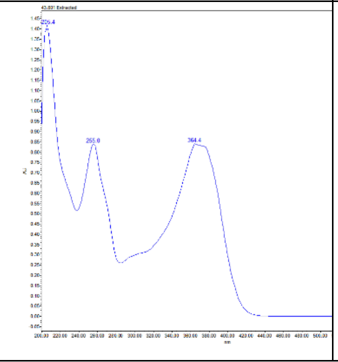	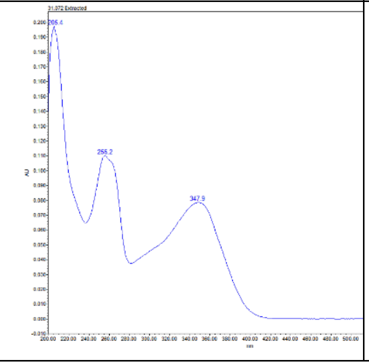	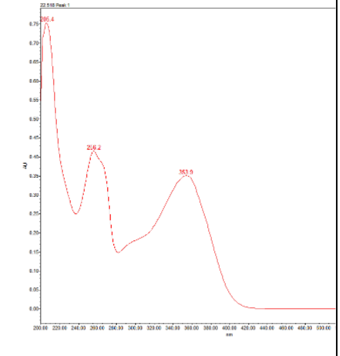		

Table S2. The concentration of the main components of *Epilobium* species, based on published data and the relevant Herb MaRS score, which is based on the potential anti-inflammatory, antiviral and antioxidant activities (*in vitro* and *in vivo*).

Compound	Activity ***in vitro*** **assay**	Known activity; ***in vivo*** **assay**	Content, mg/g	Method	CAS-Number	Herb MaRS Ranking
Afzelin (Kaempferol-3-O-rhamnoside)	Antioxidant, antitumor, anti-inflammatory (Akter et al., 2022), antibacterial, antipyretic, antivirus (HSV-1) (Zhao et al., 2021)	Anti-inflammatory (Zhao et al., 2021); anti-tumour, antioxidant (Akter et al., 2022)	1.6-3.7<br>0.89-1.25	HPLC-PDA (Monschein et al., 2015b)<br>UHPLC-DAD (Baert et al., 2017)	482-39-3	3
Avicularin (Quercetin 3-alpha-L-arabinofuranoside)	Antioxidant, anti-inflammatory, anti-allergic, anti-tumour, hepatoprotective, α-glucosidase inhibitory (Lee et al., 2019; Zhang et al., 2020)	Neuroprotective (Z. Li et al., 2024)	0.14-0.19 (leaves)	HPLC-DAD (Mykhailenko et al., 2025)	572-30-5	3
Myricetin	Anti-inflammatory, antiviral (e.g., against PRV, COVID), antioxidant, anticancer, antibacterial (Agrawal et al., 2023; Song et al., 2021)	Anti-inflammatory (Hiermann et al., 1998)	0.01-0.09;<br>0.02-1.98	HPLC-DAD (Mykhailenko et al., 2025)	529-44-2	5
Isomyricetrin (Myricetin 3-O-β-D-glucoside)	Anti-inflammatory, antiviral (e.g., against PRV, COVID), antioxidant, anticancer, antibacterial (Agrawal et al., 2023; Song et al., 2021)	Anti-inflammatory (Hiermann et al., 1998)	0.01-0.09;<br>0.02-1.98	HPLC-DAD (Mykhailenko et al., 2025)	529-44-2	3

Guaijaverin (Quercetin-3-O-α-L-arabinopyranoside)	Anti-inflammatory, antioxidant, antimicrobial, antiplaque (Prabu et al., 2006)	Antiinflammatory, antiallergenic (Park et al., 2024)	0.26-0.59 (leaves)	HPLC-DAD(Mykhailenko et al., 2025)	22255-13-6	3
Quercetin	Anti-proliferative, antioxidative, neuroprotective, anti-inflammatory, pleiotropic kinase and α-glucosidase inhibitor	Anti-inflammatory, anti-apoptotic, cardioprotector (Guo et al., 2025)	0.01 – 0.110<br>1.50<br>0.07	UPLC-MS/MS (Agnieszka et al., 2018a)<br>HPLC-PDA (Monschein et al., 2015b)<br>HPLC-UV (Maruška et al., 2014)	6151-25-3	3
Quercitrin (Quercetin-3-O-rhamnoside)	Anti-inflammatory	Anti-inflammatory (Comalada et al., 2005)	1.64<br>1.5-3.4	HPLC-UV (Maruška et al., 2014)<br>HPLC-PDA (Monschein et al., 2015b)	522-12-3	3
Hyperoside (Quercetin-3-O-galactosid)	Anti-tumour, antifungal, anti-inflammatory, anti-viral (HBV) (Wu et al., 2007), influenza virus, anti-oxidative; antidepressant, neuroprotective (Song & Lin, 2025)	Anti-inflammatory (Ku et al., 2015; Wu et al., 2007)	4.59<br>0.3-0.7	HPLC-UV (Maruška et al., 2014)<br>HPLC-PDA (Monschein et al., 2015b)	482-36-0	5
Isoquercitrin (Quercetin-3-O-glucoside)	Anti-inflammatory, antiviral, neuroprotective, antioxidant, anti-proliferative	Anti-inflammatory (Comalada et al., 2005); neuroprotective (Yang et al., 2021), cardiotoxicity (Wang et al., 2024)	0.0156<br>1.40–3.08	HPLC-ESI-QTOF-MS/MS (Kowalik et al., 2022)<br>HPLC-DAD (Mykhailenko et al., 2025)	482-35-9	5
Rutin (Quercetin-3-O-rutinoside)	Anti-inflammatory, antidiabetic, antioxidant, neuroprotective, nephroprotective, hepatoprotective, reducing Aβ oligomer	Anti-inflammatory (Yoo et al., 2014)	0.046 – 0.120<br>0.531 – 0.473	UPLC-MS/MS (Agnieszka et al., 2018a)<br>HPLC-UV (Lasinskas et al., 2020)	153-18-4	3
Gallic acid	Antimicrobial, antioxidant, antimicrobial, anti-inflammatory, anticancer, antiviral (H1N1)(You et al., 2018), SARS-CoV-2 (Lin et al., 2022)	Anti-inflammatory (Arinno et al., 2025)	0.012 – 0.070<br>0.101 – 0.120	UPLC-MS/MS (Agnieszka et al., 2018a)<br>HPLC-UV (Lasinskas et al., 2020)	149-91-7	5
Ellagic acid	Antioxidant; drug-metabolizing enzymes; analgesic, antimicrobial, antiproliferative, influence on xenobiotic metabolism	Anti-inflammatory (Lin et al., 2020)	1.024-1.892<br>10.521-25.905	UPLC-MS/MS (Agnieszka et al., 2018a)<br>HPLC-UV (Lasinskas et al., 2020)	476-66-4	3
Oenothein B	Antioxidant, anti-inflammatory, antifungal, anti-virus (HCV) (Tamura et al., 2019), antitumor against MM2 ascites tumour, inhibitor of poly(ADP-ribose) glycohydrolase	Antitumor (Miyamoto et al., 1993); anti-inflammatory (Li et al., 2026); angiogenic (Silva et al., 2024)	2.82- 3.56<br>41.77 – 72.91<br>6.117 – 14.422	HPLC-DAD (Agnieszka et al., 2018a)<br>HPLC-DAD-MS (Granica et al., 2012)<br>HPLC-UV (Lasinskas et al., 2020)	104987-36-2	5
Chlorogenic acid	inhibits the production of TNF-α, IL-1β, suppresses iNOS and COX-2 protein expression (Huang et al., 2023)	Anti-inflammatory (Q. Q. Li et al., 2024)	0.02-4.71	UHPLC-DAD (Baert et al., 2017)	906-33-2	1

a* The ranking score ranges from 0 to 5, with 0 being the least and 5 being the most suitable. 0 = has reported bioactivity but not directly related to antivirus or anti-inflammatory activity. Rankings based on pharmacological relevance to anti-inflammatory, antiviral, antioxidant activities, concentration, and literature support

Table S3. SMILEs files of phenolic compounds from *Epilobium* species

Compound	SMILES	ChEMBL number	PubChem BioAssay
Afzelin	C[C@H]1[C@@H]([C@H]([C@H]([C@@H](O1)OC2=C(OC3=CC(=CC(=C3C2=O)O)O)C4=CC=C(C=C4)O)O)O)O	CHEMBL240528	180
Avicularin	C1=CC(=C(C=C1C2=C(C(=O)C3=C(C=C(C=C3O2)O)O)O[C@H]4[C@@H]([C@H]([C@@H](O4)CO)O)O)O)O	CHEMBL471282	24
Myricetin	C1=C(C=C(C(=C1O)O)O)C2=C(C(=O)C3=C(C=C(C=C3O2)O)O)O	CHEMBL164	215
Isomyricetrin	C1=C(C=C(C(=C1O)O)O)C2=C(C(=O)C3=C(C=C(C=C3O2)O)O)O[C@H]4[C@@H]([C@H]([C@@H]([C@H](O4)CO)O)O)O	CHEMBL2282026	26
Guaijaverin	C1[C@@H]([C@@H]([C@H]([C@@H](O1)OC2=C(OC3=CC(=CC(=C3C2=O)O)O)C4=CC(=C(C=C4)O)O)O)O)O	CHEMBL464507	39
Quercetin	C1=CC(=C(C=C1C2=C(C(=O)C3=C(C=C(C=C3O2)O)O)O)O)O	CHEMBL50	2947
Quercitrin	C[C@H]1[C@@H]([C@H]([C@H]([C@@H](O1)OC2=C(OC3=CC(=CC(=C3C2=O)O)O)C4=CC(=C(C=C4)O)O)O)O)O	CHEMBL82242	182
Hyperoside	C1=CC(=C(C=C1C2=C(C(=O)C3=C(C=C(C=C3O2)O)O)O[C@H]4[C@@H]([C@H]([C@H]([C@H](O4)CO)O)O)O)O)O	CHEMBL251254	95
Isoquercitrin	C1=CC(=C(C=C1C2=C(C(=O)C3=C(C=C(C=C3O2)O)O)O[C@@H]4[C@H]([C@@H]([C@H]([C@@H](O4)CO)O)O)O)O)O	CHEMBL250450	181
Rutin	C[C@H]1[C@@H]([C@H]([C@H]([C@@H](O1)OC[C@@H]2[C@H]([C@@H]([C@H]([C@@H](O2)OC3=C(OC4=CC(=CC(=C4C 3=O)O)O)C5=CC(=C(C=C5)O)O)O)O)O)O)O)O	CHEMBL226335	283
Gallic acid	C1=C(C=C(C(=C1O)O)O)C(=O)O	CHEMBL288114	966
Ellagic acid	C1=C2C3=C(C(=C1O)O)OC(=O)C4=CC(=C(C(=C43)OC2=O)O)O	CHEMBL6246	481
Oenothein B	C1C2C3C(C(C(O2)O)OC(=O)C4=CC(=C(C(=C4OC5=C(C(=C6C(=C5)C(=O)OCC7C(C(C(C(O7)O)OC(=O)C8=CC(=C(C(=C8OC9=C(C(=C(C(=C9)C(=O)O3)C2=C(C(=C(C=C2C(=O)O1)O)O)O)O)O)O)O)O)OC(=O)C1=CC(=C(C(=C1)O)O)O)OC(=O)C1=CC(=C(C(=C1 6)O)O)O)O)O)O)O)O)OC(=O)C1=CC(=C(C(=C1)O)O)O	CHEMBL505179	30
Chlorogenic acid	C1[C@H]([C@H]([C@@H](C[C@@]1(C(=O)O)O)OC(=O)/C=C/C2=CC(=C(C=C2)O)O)O)O	CHEMBL284616	173
